# Allosteric control of hemoglobin S fiber formation by oxygen and its relation to the pathophysiology of sickle cell disease

**DOI:** 10.1073/pnas.1922004117

**Published:** 2020-06-11

**Authors:** Eric R. Henry, Troy Cellmer, Emily B. Dunkelberger, Belhu Metaferia, James Hofrichter, Quan Li, David Ostrowski, Rodolfo Ghirlando, John M. Louis, Stéphane Moutereau, Frédéric Galactéros, Swee Lay Thein, Pablo Bartolucci, William A. Eaton

**Affiliations:** ^a^Laboratory of Chemical Physics, National Institute of Diabetes and Digestive and Kidney Diseases, National Institutes of Health, Bethesda, MD 20892-0520;; ^b^Laboratory of Molecular Biology, National Institute of Diabetes and Digestive and Kidney Diseases, National Institutes of Health, Bethesda, MD 20892-0520;; ^c^Department of Genetics, Hôpital Henri Mondor, Assistance Publique-Hôpitaux de Paris, Université Paris-Est Créteil, 94010 Créteil, France;; ^d^IMRB Team 2, GRex, 94010 Créteil, France;; ^e^Sickle Cell Referral Center, Hôpital Henri Mondor, Assistance Publique-Hôpitaux de Paris, Université Paris-Est Créteil, 94010 Créteil, France;; ^f^Sickle Cell Branch, National Heart, Lung, and Blood Institute, National Institutes of Health, Bethesda, MD 20892

**Keywords:** sickle cell, protein fibers, polymerization

## Abstract

The pathology of sickle cell disease is caused by polymerization of the abnormal hemoglobin S upon deoxygenation in the tissues to form fibers in red cells, causing them to deform and occlude the circulation. Drugs that allosterically shift the quaternary equilibrium from the polymerizing T quaternary structure to the nonpolymerizing R quaternary structure are now being developed. Here we update our understanding on the allosteric control of fiber formation at equilibrium by showing how the simplest extension of the classic quaternary two-state allosteric model of Monod, Wyman, and Changeux to include tertiary conformational changes provides a better quantitative description. We also show that if fiber formation is at equilibrium in vivo, the vast majority of cells in most tissues would contain fibers, indicating that it is unlikely that the disease would be survivable once the nonpolymerizing fetal hemoglobin has been replaced by adult hemoglobin S at about 1 y after birth. Calculations of sickling times, based on a recently discovered universal relation between the delay time prior to fiber formation and supersaturation, show that in vivo fiber formation is very far from equilibrium. Our analysis indicates that patients survive because the delay period allows the majority of cells to escape the small vessels of the tissues before fibers form. The enormous sensitivity of the duration of the delay period to intracellular hemoglobin composition also explains why sickle trait, the heterozygous condition, and the compound heterozygous condition of hemoglobin S with pancellular hereditary persistence of fetal hemoglobin are both relatively benign conditions.

## Significance

The root cause of pathology in sickle cell disease is the polymerization of the mutant hemoglobin S upon deoxygenation in the tissues to form fibers. Both the amount of fiber at equilibrium and the kinetics of fiber formation depend on the partial pressure of oxygen. We show that control of polymerization by oxygen at equilibrium can be better explained by a recent extension of the famous two-state allosteric model of Monod, Wyman, and Changeux. Because of the unusual kinetics, polymerization is far out of equilibrium, which explains why patients with the disease manage to survive, while those with high levels of fetal hemoglobin and those with sickle trait (the heterozygous condition) have relatively benign conditions.

A single mutation from A to T in the β globin gene of hemoglobin, resulting in replacement of negatively charged glutamic acid by hydrophobic valine on the molecular surface, is responsible for sickle cell disease. The amino acid change causes polymerization of this mutant hemoglobin (hemoglobin S [HbS]) to form fibers upon deoxygenation in the tissues, the root cause of the pathology of the disease. The fibers make the red blood cells less flexible and distort the shape of the cells, a process typically referred to as sickling. The decreased flexibility results in occlusion of the small vessels, which deprives the tissues of oxygen, causing sporadic episodes of pain so severe that they are called sickle cell crises. The repeated sickling/unsickling cycles cause the sickle red cells to become extremely fragile with a half-life one-sixth that of a normal red cell (1), resulting in a chronic hemolytic anemia. The repeated vaso-occlusion and chronic anemia underlie the chronic organ damage, and no organ is spared in sickle cell disease.

Basic research on sickle cell disease has had a long and interesting history. The 1949 landmark work of the legendary genius of 20th century chemistry, Linus Pauling, showed that sickle cell anemia is not only the first human disease to be understood at a molecular level (the first molecular disease) (2, 3), it is also the first disease known to be caused by protein aggregation. Consequently, the methods and results of research on HbS, especially the mechanism of fiber formation, have become a paradigm for current research on protein and peptide aggregation mechanisms associated with neurodegenerative diseases such as Alzheimer’s and Parkinson’s disease (4–8).

**Fig. 1.**
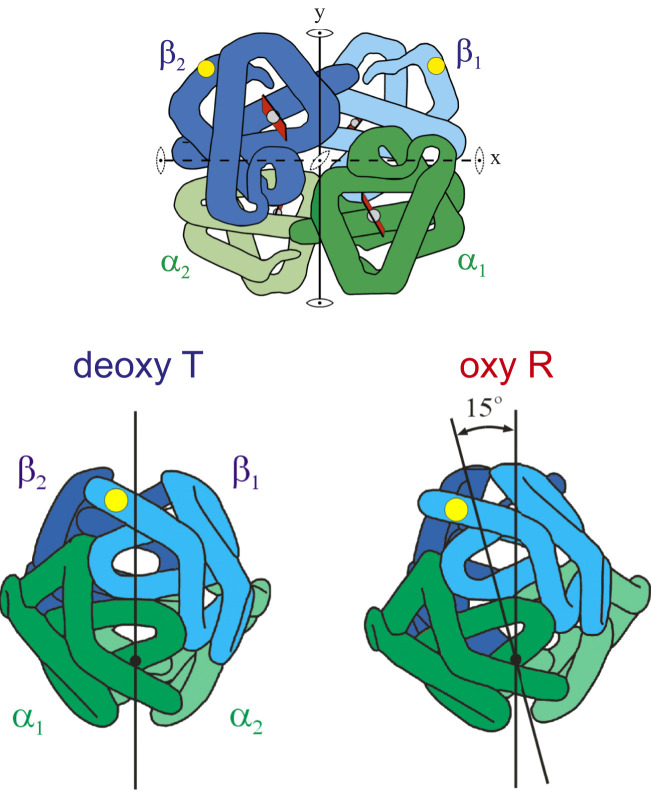
Cartoons of hemoglobin structure. (*Top*) The schematic structure shows the location of the amino acid replacement of glutamate with valine on the molecular surface at the sixth position in the sequence from the N terminus of the β chains (yellow dots). (*Bottom*) Cartoons show that the difference in quaternary structure between oxyhemoglobin and deoxyhemoglobin consists primarily of a 15° relative rotation of the two αβ dimers related by a twofold rotation axis. Adapted with permission from ref. 14.

Pauling became interested in sickle cell disease when the noted hematologist, William B. Castle, told him about the basic observation that red cells from patients with sickle cell disease are sickled in the venous blood but not in the arterial blood (9). Castle also probably described the experiments of a Johns Hopkins medical student, Irving J. Sherman, who reported that deoxygenated sickle cells exhibit birefringence, which disappears upon reoxygenation (10). Pauling was quite interested in the properties of hemoglobin (11–13) and realized from Sherman’s observation of birefringence that it must be due to aggregation of the hemoglobin to form an ordered structure in the red cells. In his famous paper showing the difference in electrophoretic pattern of normal and sickle hemoglobin, he reasoned that for deoxygenated HbS, but not oxygenated HbS, to aggregate, the amino acid replacement is on the molecular surface and that oxygenated and deoxygenated HbS molecules must have different conformations (2). It is, of course, well known that Pauling’s brilliant predictions concerning the amino acid change and differences in conformation between oxygenated and deoxygenated hemoglobin turned out to be correct (Fig. 1) (15–17). However, Pauling left several important questions unanswered that have motivated many basic research advances over the past 70 y. The most important biophysical advances include the construction of a molecular-resolution structural model for the fiber (18–23), a thorough description of the thermodynamics (24–30), the kinetics and mechanism of fiber formation (31–35), and approaches to drug therapy based on these findings (36, 37).

Given the recent resurgence of interest in finding new drugs for sickle cell disease (37) and attempts to treat the disease with drugs that reduce sickling by shifting the quaternary equilibrium toward the nonpolymerizing R conformation (37, 38–42), it seems timely to update what we now know on how fiber formation is controlled by oxygen pressure and its relation to the pathophysiology of the disease. In this work we show that while the quaternary two-state allosteric model of Monod, Wyman, and Changeux (MWC) (30, 43) with no adjustable parameters provides a remarkably good description of the equilibrium data on fiber formation, a quantitatively better description is provided by applying the tertiary two-state allosteric model (44–47), which extends the MWC model to include tertiary conformational equilibria. Using a recently discovered universal supersaturation relation that connects kinetics and equilibria (48), we also show that fiber formation is very far from equilibrium in vivo, which strengthens the case that it is the unusual fiber formation kinetics that make the disease survivable (31, 37, 49, 50). The kinetics further explain why both sickle trait, the heterozygous condition in which the intracellular concentration of HbS is <50%, and the compound heterozygous condition of HbS with hereditary persistence of fetal hemoglobin (S/HPFH), in which the intracellular HbS is diluted with fetal hemoglobin (HbF), are both relatively benign.

## Results

### Determination of Solubility from Oxygen Binding Curves.

**Fig. 2.**
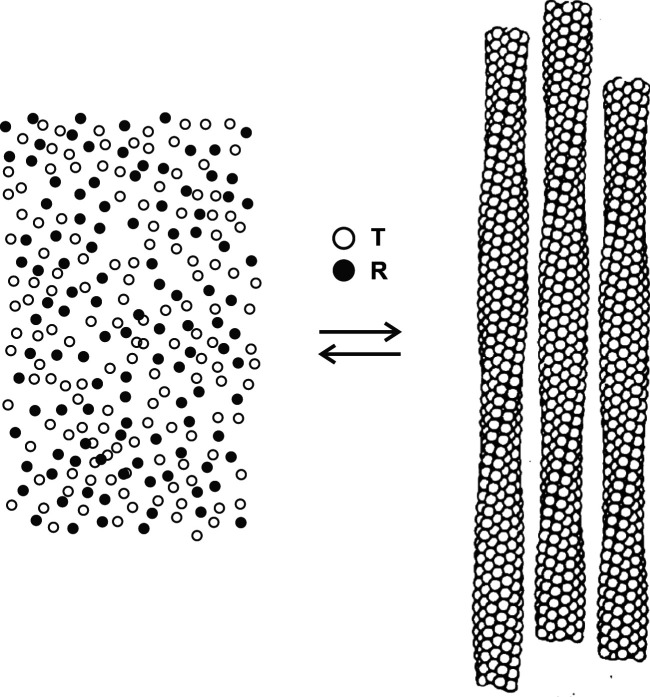
Schematic structure of gel with HbS partially saturated with oxygen showing that only the T quaternary structure enters the fiber. The total concentration of free HbS tetramers (*Left*) is the solubility, which is an accurate measure of the thermodynamic stability of the fiber (*Right*).

The thermodynamic stability of the fiber as a function of the fraction of the four Hb subunits with oxygenated hemes (fractional saturation between 0 and 1) was determined many years ago from careful solubility measurements (30). In these experiments the temperature of an HbS solution at partial saturation with oxygen was increased from 0 °C, where the solution is liquid, to 23.5 °C. After a delay, fibers formed at the elevated temperature, resulting in a viscous solution, called a gel. The gel was then subjected to ultracentrifugation, which sediments the fibers, and allowed optical absorption measurements in the near infrared spectral region (700 to 1,100 nm) to determine both the total HbS concentration and fractional saturation with oxygen of the unpolymerized HbS in the liquid supernatant. Because the gel consists of just two phases, a liquid phase and a fiber-containing polymer phase, the concentration of HbS in the liquid supernatant corresponds to the solubility and is a direct measure of the thermodynamic stability of the fiber (Fig. 2) (27).

**Fig. 3.**
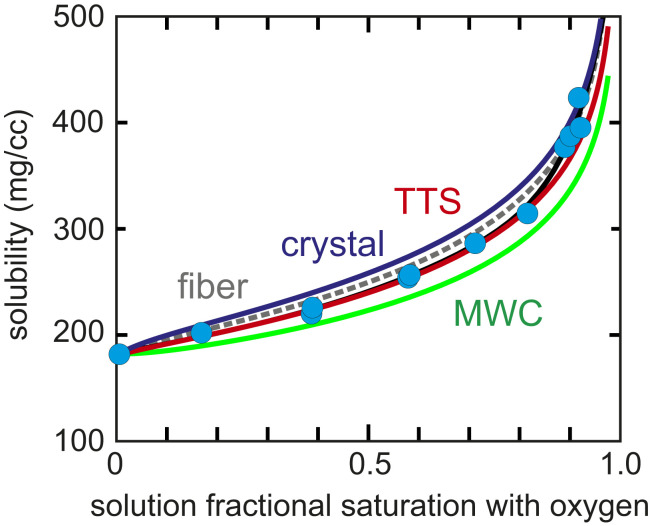
Measured solubility of HbS as a function of the fractional saturation with oxygen of the tetramers in the liquid phase with oxygen and theoretically predicted values based on oxygen binding curves. The filled cyan circles are the measured solubilities. Dashed gray (fiber) curve is solubility calculated from the free tetramer cooperative binding curve (Eq. **3**) and the measured noncooperative fiber binding curve (30) (*K*_*P*_ = 0.0059 torr^−1^) using Eq. **1**. The free tetramer cooperative binding curve is taken from the measurements of Gill et al. (51) at 25 °C using the MWC saturation function (43) (Eq. **3**) with *L* = 60,500, *K*_*R*_ = 1.47 torr^−1^, and *K*_*T*_ = 0.016 torr^−1^. Dark blue (crystal) curve is solubility calculated from the free tetramer cooperative binding curve (Eq. **3**) and the binding curve of the HbA single crystal in T quaternary structure (Eq. **4**) at 25 °C (52) (*K*_*P*_ = *K*_*crystal*_ = 0.0036 torr^−1^) using Eq. **1**. Green (MWC) curve is solubility calculated from the free tetramer cooperative binding curve (Eq. **3**) and the noncooperative binding curve of the fiber assumed to have the same affinity as the free tetramer in the T quaternary structure in the liquid phase with affinity *K*_*T*_ = 0.016 torr^−1^ (Eq. **5**). Red (TTS) curve is solubility calculated from the free tetramer cooperative binding curve (Eq. **3**) and the best least squares fit to the data points using Eq. **6** of the TTS model [with the known 25 °C parameters of *K*_*t*_ = 0.0036 torr^−1^ (52), *K*_*r*_ = 3.7 torr^−1^ (47)] with one adjustable parameter, *l*_*t*_ = 840. In this fit, the points at saturations greater than 0.85 are the most uncertain and were downweighted by a factor 10 relative to the points at lower saturation. Black curve is an empirical fit to the data points.

Fig. 3 shows the data of Sunshine et al. (30) on solubility as a function of the fractional saturation with oxygen of HbS in the liquid phase, which remains the most accurate equilibrium data to date. Also shown are the predicted solubilities based on the polyphasic thermodynamic linkage relation of Gill and Wyman between solubility and oxygen binding that satisfies the Gibbs–Duhem relation for a three-component system of hemoglobin, oxygen, and aqueous solvent (53). The solubility (*c*_*s*_) at each oxygen pressure (*p*) is related to the fractional saturation of the hemoglobin in the liquid (*y*_*s*_) and polymer phases (*y*_*P*_) by (29, 54)

$$\gamma_{s}c_{s}=\gamma_{s}^{0}c_{s}^{0}\exp\left(4\int_{0}^{p}\frac{y_{s}-y_{P}}{\left(1-\left[\left(1/c_{p}-v\right)/\left(1/c_{s}-v\right)\right]\right)x}dx\right)\text{,}$$ [1]

where *a*_*s*_ = *γ*_*s*_*c*_*s*_ is the activity of the free HbS tetramers in the liquid phase at pressure *p*, *γ*_*s*_ is the activity coefficient at concentration *c*_*s*_, $a_{s}^{0}=\gamma_{s}^{0}c_{s}^{0}$ is the activity of the free tetramers at zero oxygen pressure, $\gamma_{s}^{0}$ is the activity coefficient at concentration $c_{s}^{0}$, *c*_*p*_ is the concentration of hemoglobin in polymer phase (= 0.69 g/mL), and *v* is the partial specific volume of hemoglobin, which we have determined to be 0.76 mL/g at the high concentration of hemoglobin found in the red cell (*SI Appendix*). Large activity coefficients result from excluded volume effects in these concentrated protein solutions and are accurately given by the virial expansion for hard spheres with a volume of 0.79 mL/g that fits the sedimentation equilibrium data (54, 55) (presumably slightly higher than the partial specific volume because the hard sphere volumes includes a hydration layer):

$$\gamma =\exp\left(8Vc+15V^{2}c^{2}+24.5V^{3}c^{3}+35.3V^{4}c^{4}+47.4V^{5}c^{5}+65.9V^{6}c^{6}\right).$$ [2]

The term (1/*c*_*p*_ − *v*)/(1/*c*_*s*_ − *v*) corresponds to the number of moles of water per mole of hemoglobin in the polymer phase divided by the number of moles of water per mole of hemoglobin in the liquid phase (53).

The free tetramers of HbS and normal hemoglobin (HbA) have identical cooperative oxygen binding curves, which can be very accurately represented by the MWC saturation function using the Adair parameters of Gill to generate the binding curve at conditions [0.15 M potassium phosphate, pH 7.2, 25 °C (51)] almost identical to those of the solubility measurements [0.15 M potassium phosphate, pH 7.0, 23.5 °C (30)].

$$y_{s}=\frac{LK_{T}p{\left(1+K_{T}p\right)}^{3}+K_{R}p{\left(1+K_{R}p\right)}^{3}}{L{\left(1+K_{T}p\right)}^{4}+{\left(1+K_{R}p\right)}^{4}}.$$ [3]

The fiber binds oxygen noncooperatively (30), so its saturation function is simply

$$y_{P}=\frac{K_{P}p}{1+K_{P}p}.$$ [4]

The fiber binding curve was obtained from the linear dichroism of a gel in which the free HbS tetramers in the corresponding supernatant (i.e., liquid phase) had a measured saturation with oxygen, to determine the oxygen pressure from Eq. **3** (30). The linear dichroism is due only to the fibers as the tetramers free in solution have no preferred orientation and, therefore, cannot contribute to the observed linear dichroism [the low affinity of the fiber is the basis of a recently developed assay for determining the fraction of sickle cells containing a significant amount of polymerized hemoglobin from single-cell oxygen affinity measurements (56)]. Unfortunately, the experimental uncertainties in the measured solubilities were not definitively determined. However, the agreement of duplicate measurements of the most accurate solubilities (i.e., below *y*_*s*_ = 0.85) indicates that the uncertainties at *y*_*s*_ < 0.85 are comparable to the width of the circles. Since Eq. **1** is exact and the activity coefficients are known, the good agreement of the solubilities with the values calculated from the free tetramer and measured fiber binding curve using Eq. **1** (dashed gray curve) also suggest that the errors cannot be much larger than the plotted width of the circles for the data points at *y*_*s*_ < 0.85.

### Theoretical Calculation of Solubility from MWC and TTS Allosteric Models.

To understand the origin of the low affinity of the fiber used in the calculation of the solubility from Eq. **1**, it is instructive to consider the predictions of the two principal allosteric models: the MWC quaternary two-state model and the tertiary two-state (TTS) allosteric model, which is an extension of the MWC model (43) to include tertiary equilibria within each quaternary structure (44, 47). The simplest assumption in applying these models is to postulate that only hemoglobin in the T quaternary structure can enter the fiber, as suggested by structural modeling, which shows that it is highly unlikely that the oxy (R) quaternary structure can enter the fiber because of multiple steric clashes (57, 58). With this assumption the saturation function for the fiber according to the MWC model is simply

$$y_{P}\left(\text{MWC}\right)=\frac{K_{T}p}{1+K_{T}p},$$ [5]

where *K*_*T*_ = 0.016 is the value for the free hemoglobin tetramers (30, 51). Fig. 3 shows that the MWC model (green curve) does a remarkably good job of reproducing the solubility data without any adjustable parameters since *K*_*T*_ is simply the binding constant for the first oxygen molecule from measurements at the lowest pressures. However, the calculated solubility yields systematically lower values.

The solubility data can be more quantitatively reproduced using the TTS allosteric model, again assuming that only the T quaternary structure polymerizes. In this case, the saturation function is

$$y_{P}\left(\text{TTS}\right)=\frac{\left(\frac{l_{T}K_{t}+K_{r}}{1+l_{T}}\right)p}{1+\left(\frac{l_{T}K_{t}+K_{r}}{1+l_{T}}\right)p},$$ [6]

where *K*_*t*_ and *K*_*r*_ are the binding constants for the *t* and *r* tertiary structures and the equilibrium constant *l*_*T*_ is the *t*/*r* population ratio in the T quaternary structure with no oxygen molecules bound. The TTS model postulates that subunits within each quaternary structure have only two conformations, a low-affinity *t* conformation and a high-affinity *r* conformation; that the affinity of the *t* conformation is the same in T and R; and, similarly, that the affinity of the *r* conformation is the same in T and R (44). Oxygenation and R bias the equilibrium population toward *r*, while deoxygenation and T bias the population toward *t*. The bias of the quaternary structures results in a *t*/*r* population ratio (*l*_*T*_) in T that is much greater than this population ratio (*l*_*R*_) in R. The TTS extension of the MWC model was required to explain the discovery of R-like CO binding rates in T (45) and T-like CO binding rates in R (46). In contrast to the MWC model, which has only a single parameter (*K*_*T*_) for oxygen binding to the T quaternary structure, the TTS model has three: *K*_*t*_, *K*_*r*_, and *l*_*T*_. The values of *K*_*t*_ and *K*_*r*_ are known from binding measurements for the T quaternary structure in a single crystal (52) (*K*_*t*_ = 0.0036 torr^−1^), conditions for which the tertiary conformation in oxygenated T remains *t*, and encapsulated in a silica gel (*K*_*r*_ = 3.7 torr^−1^), where the conformation remains in R when deoxygenated (47, 59). So fitting the solubility curve with the TTS saturation function requires a single adjustable parameter, *l*_*T*_. The red curve in Fig. 3 was calculated from Eq. **1** using the MWC saturation function (Eq. **3**) for *y*_*s*_ and Eq. **6** for *y*_*P*_. The value of *l*_*T*_ that yields the best fit is 840. This value is higher than *l*_*T*_ ∼ 200 for the T quaternary structure in solution in the absence of allosteric effectors (47) due to intertetramer interactions in the fiber. These interactions are weaker than those in the deoxyHb A crystal, presumably because of fewer intermolecular contacts for tetramers on the surface of the fiber. In the crystal, the subunits remain in the *t* tertiary conformation upon oxygen binding, so *l*_*t*_ is larger [∼10^5^ (47)] than in the fiber. However, *l*_*t*_ is sufficiently large that Eq. **6** reduces to Eq. **4** with a binding constant of *K*_*t*_.

### Calculation of Solubility in Mixtures of HbS with Normal or Fetal Hemoglobins.

Calculation of solubilities (*c*_*s*_) for the mixtures of HbS with normal (HbA) or fetal (HbF) hemoglobins as a function of fractional saturation is accomplished by solving the following two simultaneous mass conservation equations (derived in *SI Appendix*) for the two unknowns *x*_*βS*_ (fraction of β^S^ chains) and *c*_*s*_, the quantity we seek:

$$\begin{array}{l} x_{\beta S}c_{s}\left(c_{p}-c_{0}\right)+\Gamma Z\frac{c_{s}}{c_{s}^{0}}\left(e_{1}x_{\beta S}^{2}+e_{2}x_{\beta S}\left(1-x_{\beta S}\right)\right)c_{p}\left(c_{0}-c_{s}\right)=X_{\beta S}c_{0}\left(c_{p}-c_{s}\right)\\ \left(1-x_{\beta S}\right)c_{s}\left(c_{p}-c_{0}\right)+\Gamma Z\frac{c_{s}}{c_{s}^{0}}\left(e_{2}x_{\beta S}\left(1-x_{\beta S}\right)\right)c_{p}\left(c_{0}-c_{s}\right)=\left(1-X_{\beta S}\right)c_{0}\left(c_{p}-c_{s}\right), \end{array}$$ [7]

with the definitions

$$Z\equiv \frac{L{\left(1+K_{P}p\right)}^{4}}{L{\left(1+K_{T}p\right)}^{4}+{\left(1+K_{R}p\right)}^{4}}\;\text{and}\;\Gamma \equiv \frac{\gamma_{s}}{\gamma_{s}^{0}}{\left(\frac{a_{H_{2}O}}{a_{H_{2}O}^{0}}\right)}^{n},$$ [8]

where *X*_*βS*_ is the total fraction of β^S^ chains (= HbS) in the sample, (1 − *X*_*βS*_) is the total fraction of β^A^ (= HbA) or γ (= HbF) chains in the sample, *x*_*βS*_ is the fraction of β^S^ chains in the solution phase, $a_{H_{2}O}^{o}$ is the activity of the water in the supernatant for pure HbS, $a_{H_{2}O}$ is the activity of the water at the solubility for the mixture, and *n* (= 2,500) is the number of moles of water per mole of hemoglobin in the polymer phase. The polymerization probability, *e*_1_, for the α_2_β_2_^S^ homotetramer is 1; *e*_2_ is the copolymerization probability of either the α_2_β^S^β^A^ or the α_2_β^S^γ heterotetramer; *c*_*p*_ is the hemoglobin concentration in the polymer (= 0.69 g/mL); and *c*_0_ is the total hemoglobin concentration in the sample ([HbS] + [HbA] or [HbS] + [HbF]). It is assumed that neither homotetramer, α_2_β_2_^A^ nor α_2_γ_2_, can enter the fiber. The copolymerization probability of the hybrid tetramer α_2_β^S^β^A^, *e*_2_, is 0.37 (54), while *e*_2_ for the α_2_β^S^γ heterotetramer is taken as 0.1.

### Relevance of Solubility to Pathophysiology of Sickle Cell Disease.

**Fig. 4.**
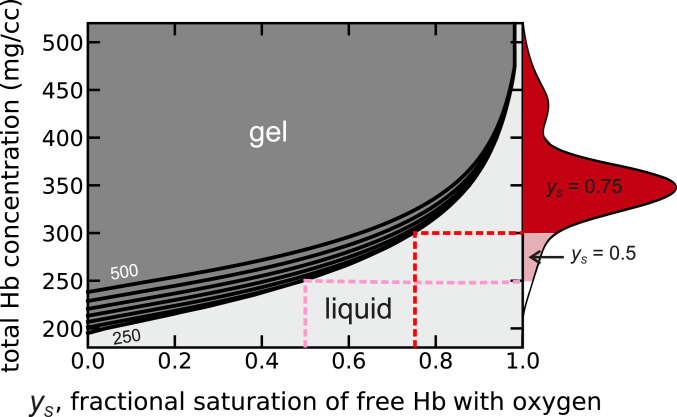
Physiological concentration–saturation phase diagram at 37 °C for Hb in red cells from homozygous sickle cell patients calculated from the average hemoglobin composition of 88% HbS and 12% (HbF + HbA2) for 29 patients. Given the absence of information on the heterogeneity in the HbF + HbA2 composition, which is certainly present, we have assigned a fixed composition of 12% to all cells. The solubility (black curves at intervals of 50 mg/cc, from 250 to 500 mg/cc) increases with increasing total Hb concentration because the species α_2_γ_2_ and α_2_β^S^γ build up in the liquid phase as the total Hb concentration increases due to lack of copolymerization of α_2_γ_2_ and only partial copolymerization of α_2_β^S^γ.The solution contains fibers at equilibrium for all values of the Hb concentration and fractional saturation of the Hb tetramers in the liquid phase above the solubility lines and no fibers below the lines. In vivo, the distribution of intracellular HbS concentrations vary from 250 to 500 mg/cc, while the oxygen pressure is less than 40 torr in most tissues (60, 61), corresponding to a hemoglobin saturation with oxygen in the liquid phase of about 75%. Solubilities were calculated from Eq. **7** with *e*_2_ = 0.1 and with *K*_*T*_ = 0.0093 torr^−1^, *K*_*R*_ = 1.04 torr^−1^, *K*_*P*_ = 0.006 torr^−1^, and *L* = 5.6 × 10^5^ in Eq. **8**. The concentration distribution on the right-hand *y* axis is the same as in Fig. 5 and is the average of the distributions for cells from 29 homozygous SS patients not being treated with hydroxyurea or transfused. The red shaded area of the concentration distribution shows that at equilibrium and 75% saturation of hemoglobin in the liquid phase the vast majority of cells would contain fibers in the tissues, while for oxygen pressures at the p50, almost every cell would contain fibers.

As shown in Fig. 4, it is useful to think of the measured solubilities as a boundary line that separates the two phases of an HbS solution at equilibrium. Solutions with HbS concentrations and saturations with oxygen that lie above the line will contain fibers at equilibrium. If a sufficient fraction of the HbS is polymerized, the increase in intracellular viscosity will make the red cells significantly less flexible. Solutions with concentrations and saturations below the line will remain liquid with no fibers at equilibrium. Red cells with these compositions have normal flexibility and can never sickle. Since cell populations have a wide distribution of intracellular hemoglobin concentrations, we can use the phase diagrams (see Figs. 4, 6, and 7) to divide the cell population into a subpopulation which, at a specified fractional saturation, would contain polymers at equilibrium and one which would not. These subpopulations are shown as the filled regions of the total intracellular hemoglobin concentration distribution shown on the *y* axis on the right side of the diagram.

**Fig. 5.**
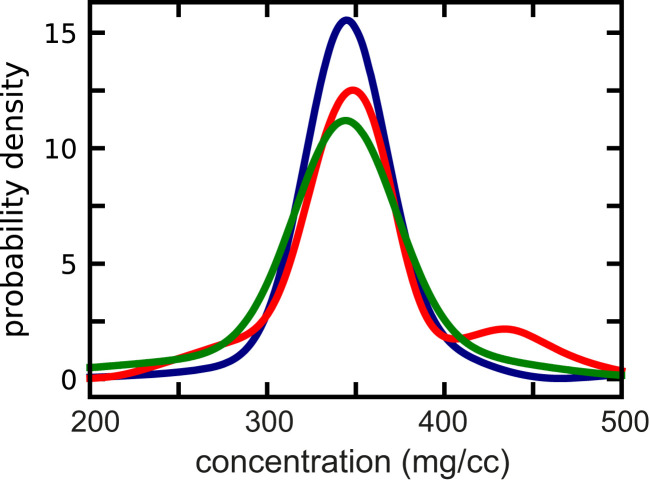
Intracellular hemoglobin concentration distributions. Blue curve shows average of 61 determinations of concentration distributions measured with the Advia 2120 for red cells from six individuals with sickle trait, which is very close to the average for 22 individuals with only normal hemoglobin (62). Red curve shows concentration distribution that is average of concentration distributions derived from density distributions for cells from 29 homozygous SS patients not treated with hydroxyurea. The derivation of concentration distributions from density distribution is described in *Methods*. Green curve shows concentration distribution determined measured with the Advia 2120 for cells from 16 homozygous SS patients being treated with hydroxyurea.

Surprisingly, there has been very little published data on the distribution of total intracellular hemoglobin concentration in patients with sickle cell (SS) disease. Fig. 5 shows the average of the distributions we measured for 16 SS patients using the Siemens Advia 2120, which determines total hemoglobin intracellular concentrations based on a dual-angle light-scattering method (63). These patients are, however, on hydroxyurea therapy. Since, for present purposes, we are primarily interested in analyzing the data for untreated SS patients, we turned to red cell density data for a group of SS patients who were not on hydroxyurea therapy and had not been transfused. Although concentration is linearly related to density, there are very large differences reported in the literature for converting red cell density to intracellular hemoglobin concentration [e.g., density/[Hb] = 3.61 at a density of 1.091 (64); density/[Hb] = 3.02 at a density of 1.090 (65)]. The most accurate conversion from density to concentration appears to be the data of Mohandas et al. (63), who used a dual light-scattering method to determine the mean intracellular concentration of density fractioned cells. However, even the small differences in the slope and intercept in a linear fit to their data are quite significant because of the enormous sensitivity of the delay time (*t*_*d*_) to hemoglobin concentration [1/*t*_*d*_ ∼ (3.61/3.02)^30^ = 200, see below]. Since the density depends on both the partial specific volumes of hemoglobin and the remaining composition of the red cell (water, salts, and other proteins), to reduce the uncertainty, we determined the partial specific volume of hemoglobin at the high concentrations found in red cells (*SI Appendix*).

The distributions of intracellular hemoglobin concentrations for SS cells shown in Fig. 5 correspond to the average of the distributions for 29 patients who at the time of the density measurements were not on hydroxyurea therapy and had not been recently transfused (red curve) and to average of the concentration distributions for 16 patients on hydroxyurea therapy (green curve) that we have measured with the Advia 2120. The concentration distributions from the density measurements for the 29 SS patients not on hydroxyurea are most relevant for the present purposes, which is to predict in vivo sickling for cells of untreated patients. The concentrations range from 250 to 500 mg/cc, but the distributions vary markedly for the different patients. The concentration distribution is broader than found for normal red cells because of two effects that result in two additional populations of cells. The destruction of damaged red cells stimulates erythropoiesis that produces release of immature red cells (reticulocytes), which have the lowest intracellular hemoglobin concentration (66), while repeated cycles of sickling and unsickling cause the loss of potassium ions and water that result in red cells with a higher concentration than found in normal blood (67) (this population contains permanently distorted cells, commonly called irreversibly sickled cells). Notice that the distributions for patients on hydroxyurea therapy do not show as many of the most concentrated cells, consistent with earlier work on hydroxyurea therapy (68).

Oxygen pressure in most human tissues is less than 40 torr (60, 61), corresponding to less than 75% saturation with oxygen (p50 = 28.5 torr, Hill *n* = 2.6). Thus, the phase diagram (Fig. 4) shows that at the oxygen saturations of hemoglobin in vivo, the concentration of hemoglobin in most cells exceeds the solubility. Consequently, if HbS polymerization were at equilibrium, almost every cell in most tissues would contain fibers. Were it not for the fact that polymerization is far out of equilibrium in vivo, as described in more detail below, patients would not survive the disease after fetal hemoglobin is replaced by adult HbS.

**Fig. 6.**
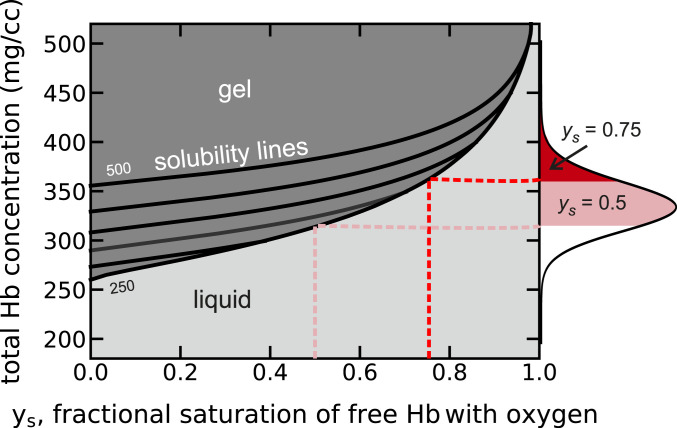
Physiological concentration–saturation phase diagram at 37 °C for Hb in red cells from sickle trait donors calculated from the average hemoglobin composition of 38% HbS and 58% HbA and 4% HbA2. The solution contains fibers at equilibrium for all values of the Hb concentration and fractional saturation of the Hb tetramers in the liquid phase above the solubility lines and no fibers below the lines. In vivo, the distribution of intracellular HbS concentrations vary from 250 to 450 mg/cc, while the oxygen pressure is less than 40 torr in most tissues (60, 61), corresponding to a hemoglobin saturation with oxygen in the liquid phase of about 75%. Solubilities were calculated from Eq. **7** with *e*_2_ = 0.37 and with *K*_*T*_ = 0.0093 torr^−1^, *K*_*R*_ = 1.04 torr^−1^, *K*_*P*_ = 0.006 torr^−1^, and *L* = 5.6 × 10^5^ in Eq. **8**. The solubility (black curves at intervals of 50 mg/cc, from 250 to 500 mg/cc) increases with increasing total Hb concentration because the species α_2_β^A^_2_ and α_2_β^S^β^A^ build up in the liquid phase as the total Hb concentration increases due to lack of copolymerization of α_2_β^A^_2_ and only partial copolymerization of α_2_β^S^β^A^. The red shaded area in the concentration distribution on the right *y* axis shows the fraction of trait cells containing fibers at equilibrium when the liquid phase is 75% saturated with oxygen, while the pink plus red shaded areas show the fraction of trait cells containing fibers when the liquid phase is 50% saturated with oxygen. The concentration distribution is the average of 61 determinations for red cells from six individuals with sickle trait.

**Fig. 7.**
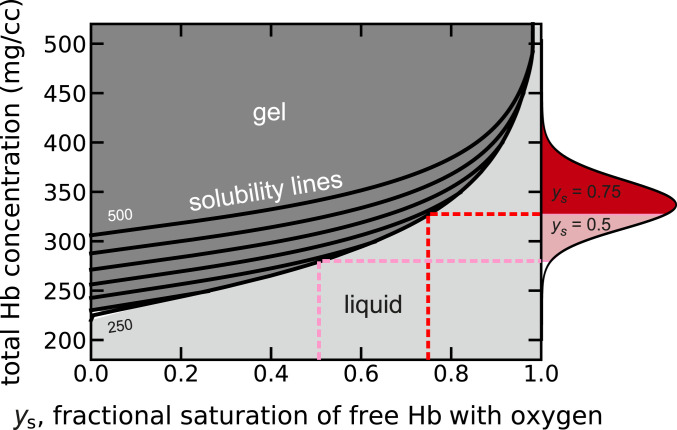
Physiological concentration–saturation phase diagram at 37 °C for a 30/70 HbF/HbS mixture as is found in S/HPFH. Solubilities were calculated from Eq. **7** with *e*_2_ = 0.1 and with *K*_*T*_ = 0.0093 torr^−1^, *K*_*R*_ = 1.04 torr^−1^, *K*_*P*_ = 0.006 torr^−1^, and *L* = 5.6 × 10^5^ in Eq. **8**. A value of 0.1 is more in keeping with composition studies, which found γ subunits in the HbS fibers (69, 70) (table 3.3 in ref. 54). The solubility (black curves at intervals of 50 mg/cc, from 250 to 450 mg/cc) increases with increasing total Hb concentration because the nonpolymerizing α_2_γ_2_ tetramer and the weakly polymerizing α_2_β^S^γ tetramer build up in the liquid phase as the total Hb concentration increases. The solution contains fibers at equilibrium for all values of the total Hb concentration and fractional saturation of the free Hb tetramers in the liquid phase above the lowest black curve. In vivo, the distributions of intracellular HbS concentrations vary from 250 to 500 mg/cc, while the oxygen pressure is less than 40 torr in most tissues (60, 61), corresponding to a hemoglobin saturation with oxygen in the liquid phase of about 75%. The red shaded area in the concentration distribution on the right *y* axis shows the fraction of S/HPFH cells containing fibers at equilibrium when the liquid phase is 75% saturated with oxygen, while the pink plus red shaded areas show the fraction of S/HPFH cells containing fibers when the liquid phase is 50% saturated with oxygen. The concentration distribution is the average of 61 determinations for red cells from six individuals with sickle trait.

The phase diagram for sickle trait in Fig. 6 shows that at 75% saturation with oxygen for the free tetramers at equilibrium a significant fraction of cells would contain fibers, while at 50% saturation a majority of cells at equilibrium would contain fibers. In the case of S/HPFH (Fig. 7), at equilibrium the majority of cells would contain fibers at 75% saturation, and at 50% saturation, almost every cell would contain fibers. Again, as discussed below, polymerization in these heterozygous conditions is far out of equilibrium, which makes both conditions relatively benign.

### Calculation of Sickling Kinetics in Vivo at Tissue Oxygen Pressures for Subjects with HbSS Sickle Cell Disease, HbAS Sickle Trait, and HbS/HPFH.

**Fig. 8.**
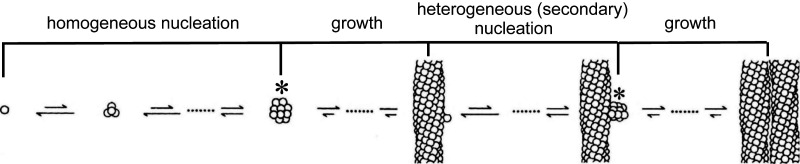
The double nucleation mechanism for HbS polymerization (33, 54, 71–73). There are two nucleated polymerization processes to HbS fiber formation, hence the name double nucleation mechanism. The first fiber in any given volume forms by the classical Oosawa nucleation growth model (74), called homogeneous because it occurs without any contact to other fibers or surfaces. The initial aggregation steps are thermodynamically unfavorable (as indicated by the relative lengths of the arrows) because the loss of translation and rotational entropy is greater than the stabilization from intermolecular contacts and the compensatory increased entropy due to low frequency intertetramer vibrations of the polymerized molecules. So the overall reaction is uphill in free energy until a critical nucleus (asterisk) is formed. Addition of molecules to the critical nucleus and all subsequent fiber growth is downhill in free energy. Except at the very highest concentrations, homogeneously nucleated fiber formation is quickly superseded by a secondary nucleation process, called heterogeneous nucleation, because fibers are nucleated on the surface of existing ones. Secondary nucleation becomes much more favorable than homogeneous nucleation from two effects. First, the free energy barrier to heterogeneous nucleation is lowered as a result of the additional stability from contacts with a fiber surface. Second, even a small reduction in the free HbS concentration from fiber formation shuts down homogeneous nucleation because of the enormous dependence of the homogeneous nucleation rate on concentration (50th to 80th powers) (34, 35). As more fibers form, there is increasing surface area for heterogeneous nucleation, providing an autocatalytic mechanism that explains the exponential growth of polymerized HbS. The exponential growth results in an apparent delay or what is often called a lag phase. Therefore, the duration of the delay period, the delay time, depends on the sensitivity of the method used to detect fiber formation.

The remarkable kinetics of HbS polymerization, with a delay period prior to the appearance of fibers that has an enormous concentration dependence, has been explained by a novel nucleation mechanism that has also been used to explain the kinetics of aggregation of the Alzheimer’s peptide (Fig. 8) (4–7). Because of these unusual kinetics, polymerization is far out of equilibrium, so the phase diagrams in Figs. 4, 6, and 7 describing the equilibrium properties do not apply to the situation in vivo. Unfortunately, there are currently no experiments on delay times for individual cells from homozygous SS patients that are deoxygenated on a physiological time scale to partial oxygen saturations of the hemoglobin. However, given a decay rate of oxygen pressure to a final saturation and an intracellular HbS concentration distribution, we can provide a partial answer to this question by theoretically calculating the fraction of cells that will sickle as a function of time.

Because the oxygen pressure is changing with time, the delay time (*t*_*d*_) is also continuously changing. The time at which fibers form inside a cell, i.e., the sickling time (*t*_*sickle*_), occurs when

$$\int_{0}^{t_{sickle}}\frac{d\tau }{t_{d}\left(\tau \right)}=1.$$ [9]

This relation of A. Szabo, derived in *SI Appendix*, shows that the sickling time (*t*_*sickle*_) is determined if the delay time (*t*_*d*_) (which is very close to the time that 10% of the equilibrium amount of polymer has formed) as a function of time (*τ*) is known. The key to this calculation is the recent discovery of a universal relation between the delay time and the supersaturation of the solution [the ratio of the total concentration of Hb to the equilibrium solubility, each multiplied by an activity coefficient (Eq. **2**)] (48). The delay time for each cell is determined by the supersaturation, which depends on the intracellular HbS concentration (Fig. 5) and the solubility as a function of the fractional saturation of HbS with oxygen (Fig. 4) that is changing with time as the saturation decreases. Knowing the solubility as a function of oxygen saturation and the universal relation between the delay time prior to fiber formation and the activity supersaturation, we can calculate the fraction of cells containing fibers (defined here as “fraction sickled”) versus time for any decay function of the oxygen partial pressure to various final values.

**Fig. 9.**
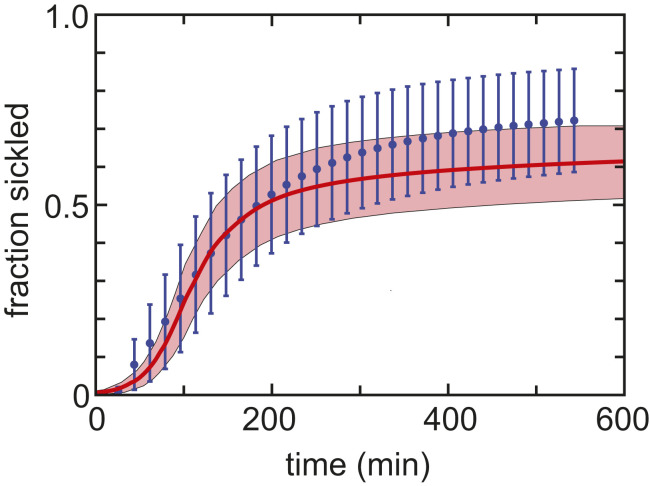
Comparison of average measured and theoretically calculated fraction SS cells sickled vs. time for slow deoxygenation to a final gas mixture of 95% nitrogen and 5% oxygen. Cells are from 16 homozygous SS patients on hydroxyurea (see *SI Appendix*, Table S1, for Hb compositions). The average composition is 79% HbS and 21% (Hb F + HbA2). The average concentration distribution is the green curve shown in Fig. 5. The measured fraction sickled vs. time shows the average fraction at each time point for 16 samples. The error bars correspond to 1 SD from the mean, which are large due to patient-to-patient differences in sickling curves that result from differences in hemoglobin composition (mainly HbF) and intracellular concentration distributions. The calculated fraction sickled vs. time was performed for each blood sample using its measured composition and total intracellular hemoglobin concentration. The pink shaded area represents the patient-to-patient variation in the calculation. *SI Appendix*, Fig. S4, shows the individual steps in the calculation of the theoretical curve.

As an important test of our calculations, we have just recently obtained an important set of data on the fraction sickled as a function of time for SS cells of known hemoglobin composition and total intracellular hemoglobin composition as the oxygen pressure is decreased, although the pressure decrease occurs on the tens of minutes time scale instead of the seconds time scale of the in vivo situation. The experiment consists of deoxygenating SS cells to a final gas mixture of 95% nitrogen and 5% oxygen in a 384-well plate format. It is the same experiment in every detail that has been used to measure sickling in trait cells, except that final oxygen pressure was 0 torr (75) instead of the 95%/5% mixture used to slow the sickling of SS cells. Fig. 9 shows the average fraction sickled for 16 HbSS patients on hydroxyurea as a function of time after starting deoxygenation with the 95%/5% nitrogen/oxygen mixture. Also shown is the theoretical calculation.

Considering that we have assumed a homogeneous distribution of both HbF and 2,3-DPG, which affects solubility and oxygen affinity (76), the agreement between experimentally measured and theoretically calculated sickling curves must be considered very good, thereby providing support for our theoretical calculations of in vivo sickling times.

The biggest unknowns for calculating the pathophysiologically relevant in vivo sickling times are the deoxygenation time, the final saturation of the red cell as it exits the narrowest vessels of the tissues (the microcirculation), and its transit time through the microcirculation where sickling can result in vaso-occlusion. There is no information on transit times in humans, but experiments on animals of different sizes show capillary transit times of 0.2 to 1.5 s in various muscles (77). However, it is now known that considerable dissociation of oxygen from red cells occurs in the precapillary arteriolar network (78, 79), so the textbook picture of oxygen delivery only occurring in the capillaries is highly oversimplified. Moreover, there is evidence that the classic picture of occlusion in the capillaries may also be oversimplified, with experiments on animals indicating that the site of occlusion is in the postcapillary venules (80) and measurements of sickled cell transit in narrow tubes suggesting that sickled cells in capillaries becoming stuck in the capillaries may not be the site of vaso-occlusion (50, 81). Consequently, to account for the total time from the onset of deoxygenation in the prearteriolar vessels to entering the venules, we have performed calculations for a range of times from 1 to 4 s. We have performed calculations where the final pressures, i.e., the pressures at the entrance to the venules, range from 0 to 50 torr. Moreover, for simplicity, we have also assumed that the oxygen pressure decreases linearly with time from 100 torr to the final pressure, as done by Karniadakis and coworkers (82).

**Fig. 10.**
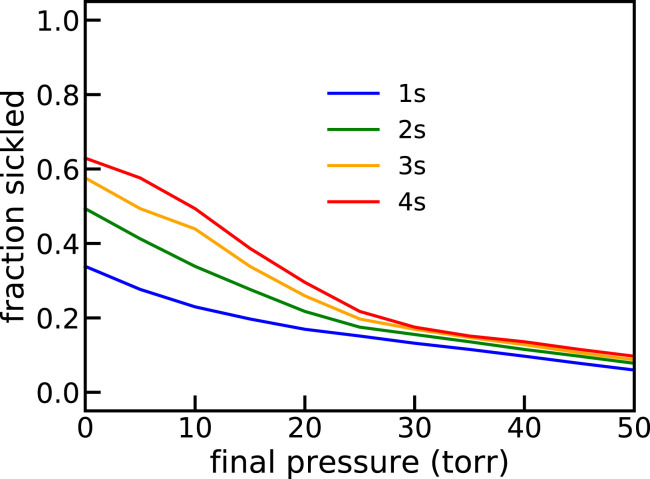
Fraction sickled as a function of final oxygen pressure after 1-, 2-, 3-, and 4-s linear decreases (ramps) for the average composition and average concentration distribution for the red cells from 29 SS patients not on hydroxyurea therapy.

*SI Appendix*, Fig. S5, shows the details of the calculation for a 1-s pressure decrease. *SI Appendix*, Fig. S6, shows the fraction sickled versus time at six different oxygen pressures. Fig. 10 shows the fraction sickled as a function of final oxygen pressure at the end of 1-, 2-, 3-, and 4-s linear decreases (ramps) for the red cells from 29 SS patients not on hydroxyurea. There are large patient-to-patient differences in both the total intracellular hemoglobin concentrations distributions and the average fraction HbF, with the result that there is a large variation in the predicted sickling kinetics. *SI Appendix*, Fig. S7, shows the distribution of fraction sickled at the end of the oxygen pressure decrease to 10, 20, and 30 torr for the 29 SS patients not on hydroxyurea.

**Fig. 11.**
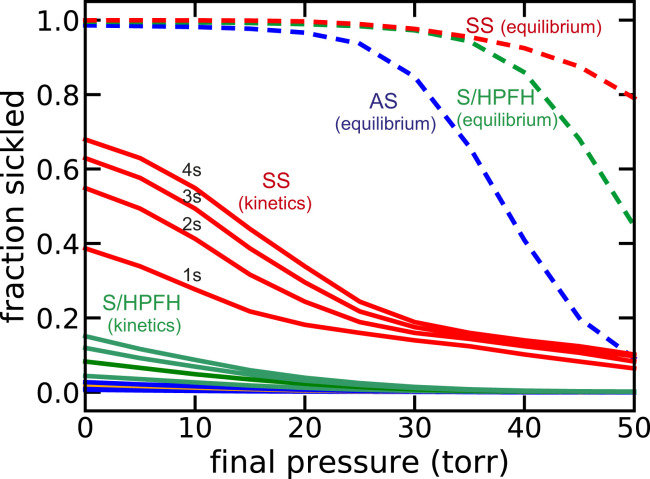
Comparison of calculated in vivo SS (red), S/HPFH (green), and AS (blue) sickling kinetics together with fraction sickled at equilibrium. Fraction sickled at the end of a 1-, 2-, 3-, and 4-s linear decrease of the oxygen pressure to pressures between 0 and 50 torr (continuous curves) and the fraction sickled at equilibrium at each pressure for cells from SS patients not being treated with hydroxyurea, sickle trait donors with average composition of 38% HbS, 58% HbA, and 4% HbA2 and for red cells having a hemoglobin composition of 30% HbF and 70% HbS as found in the compound heterozygous condition of S/HPFH. *SI Appendix*, Fig. S8, shows the fraction sickled vs. time induced by a 1-s linear decrease in oxygen pressure.

Fig. 11 compares the calculated in vivo fraction sickled at equilibrium and sickling kinetics for the three conditions: homozygous sickle cell (SS) disease, sickle trait (AS), and S/HPFH.

## Discussion

Pathophysiology (83, 84) and therapy (85) for sickle cell disease have often been discussed using an equilibrium analysis of sickling alone, but it can be highly misleading*. This is made quite clear from examination of the concentration–saturation phase diagrams in Figs. 4, 6, and 7 and the comparison of sickling at equilibrium and the kinetics of sickling in Fig. 11. The phase diagram (Fig. 4) and Fig. 11 show that in homozygous SS disease, almost every cell would be sickled in most tissues even at saturations with oxygen as high as 75%. Consequently, if fiber formation were at equilibrium, it is unlikely that patients would survive after the replacement of fetal hemoglobin with adult HbS, which is almost complete at about 1 y (1). In the case of sickle trait, the phase diagram (Fig. 6) shows that while only a small fraction of cells would sickle at 75% saturation, the majority would be sickled at 50% saturation (corresponding to an oxygen pressure of about 28 torr). Moreover, many more would be sickled in the hypertonic renal medulla. In S/HPFH, about half the cells would be sickled at equilibrium at 75% saturation, and almost every cell would be sickled at 50% saturation (Fig. 7). Consequently, the phase diagrams for sickle trait and S/HPFH suggest that both conditions would be associated with severe pathology were fiber formation at equilibrium, while both conditions are considered benign relative to sickle cell disease (85).

Oxygen binding is close to equilibrium as red cells pass through the tissues because the dissociation, binding (90), and conformation rates (44) are comparable to or faster than the transit time from the arteries to the venules. As a result, the oxygen binding measurements for normal hemoglobin measured on the tens of minutes time scale that began over 100 y ago (91) have physiological significance even though deoxygenation and reoxygenation in vivo occurs much faster. In sharp contrast, polymerization of HbS in vivo in homozygous SS disease is so far out of equilibrium because the kinetics of fiber formation for the majority of cells are slower than the transit times through the narrowest vessels of tissues. Indeed, it is the delay before fibers form that allows most cells to escape the narrow vessels before sickling occurs (Fig. 11). A similar conclusion has been reached recently from theoretical calculations by Ferrone and coworkers (92) and more recent calculations by G. Karniadakis and coworkers, which are very similar to those presented here [the Karniadakis work also includes interesting coarse-grained molecular dynamics simulations of red cell shape deformation due to fiber formation (93)]. Consequently, it is the unusual kinetics that makes the disease survivable. Moreover, both sickle trait (94) and S/HPFH (85) are relatively benign conditions because the delay times are much longer, and therefore, sickling in vivo is far less (Fig. 11).

**Fig. 12.**
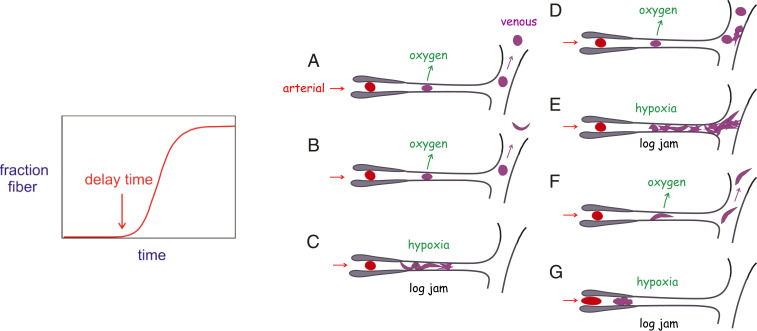
Relation between fiber formation kinetics and pathophysiology of sickle cell disease. (*Left*) Schematic of kinetic progress curve for fiber formation. The delay time is extraordinarily sensitive to HbS concentration, depending on up to 40th power of the concentration, most probably the largest concentration dependence ever observed for a chemical reaction. Thus, for example, an 8% decrease in the Hb concentration will result in a 10-fold increase in the delay time (recall for a bimolecular reaction, an 8% decrease in reactants will produce an 8% increase in the half-time for the reaction). (*Right*) Schematic of microcirculation showing an arteriole, capillary, and venule and various sickling scenarios. (*A*) The delay time is so long that the red cell squeezes through the narrow capillary before any fibers form to produce cellular distortion (sickling) and may even return without sickling all the way to the lungs, where it is reoxygenated. (*B*) Sickling occurs in larger vessel and returns to the lungs where the fibers melt and the cell unsickles. (*C*) Fibers form while the cell is in the capillary and becomes stuck, causing a log jam effect and decreased oxygen delivery to the surrounding tissue (hypoxia). (*D*) Unsickled cell escapes the both the capillary and the postcapillary venule where sickled cells are adherent to the venule endothelium. (*E*) Cell sickles and cannot escape the postcapillary venule, where sickled cells are adherent, causing a log jam (50, 81, 87). (*F*) Cell sickles in capillary but nevertheless squeezes through to the larger vessels. (*G*) Cell sickles before or within the precapillary arteriole because the concentration of intracellular hemoglobin is very high or nuclei are already present because fibers have not completely melted upon oxygenation in the lungs, enormously decreasing the delay time (88, 89).

The question for future studies is apparent from Fig. 12, which summarizes the various scenarios that may occur [although it does not include occlusion of larger vessels by collective jamming, where there has been both very informative rheological experiments and insightful theory (95, 96)]. What is the relative fraction of each of these scenarios in sickle cell patients, and how do these fractions change in sickle cell crisis? Since the transit time is a critical factor in any kinetic analysis, it raises the question of the origin of the wide range of clinical severity in sickle cell disease. Does it result primarily from patient to patient variations in sickling times or in transit times? Factors that slow the transit of red cells through the microcirculation, such as increased adherence to the vascular endothelium by red cells that are damaged form sickling/unsickling cycles or increased leukocytes associated with infection, will increase the probability of vaso-occlusion (97–99). Clinical studies comparing severity with distributions of red cell sickling times and adherence could potentially provide the answer. There are now several excellent assays for monitoring cell sickling (56, 100, 101) but not, as yet, a reliable assay for adherence.

These questions point to important experiments that are necessary for a better understanding of sickling and vaso-occlusion in vivo. From a circulatory physiology perspective these include the time dependence of oxygen pressures for humans as red cells enter and exit tissues, the transit times in humans for red cells through the smallest vessels where sickled cells could become stuck, and measurements similar to those of Ferrone and coworkers (50, 81) but which also take into account adhesion to the capillary endothelium. For more accurate calculations of sickling, information on composition heterogeneity is required, especially for the distribution of fetal hemoglobin at each total intracellular hemoglobin concentration (102). Also needed are additional measurements on purified solutions of mixtures of HbS with HbF and HbA, including delay times and solubilities as a function of fractional saturation under physiological conditions, to resolve the discrepancies in copolymerization probabilities from solubility and polymer phase composition measurements (54).

## Methods

### Red Cell Measurements.

Blood samples were collected from donors with sickle cell disease according to NIH protocols 03-H-0015 and 04-H-0161 and sickle cell trait according to NIH protocol 08-DK-004. All samples were provided as deidentified. Hemoglobin composition was determined by HPLC in the NIH Clinical Laboratory. Intracellular hemoglobin concentrations were measured with the Siemens Advia2120 Hematology System. Both experimental and calculated sickling curves shown in Fig. 9 were determined as described in Dunkelbereger et al. (34), except that the final gas composition was 95% nitrogen and 5% oxygen.

### Additional Information.

*SI Appendix* contains the derivation of the solubility equations, the conversion of density distributions to concentration distributions, the determination of the partial specific volume of hemoglobin, derivation of the equation determining the fraction sickled versus time from the time dependence of the delay time for each intracellular concentration, and the steps in the calculation of the fraction sickled versus time.

### Data Availability.

All data not contained in the main text or in *SI Appendix*, such as the original fraction versus density curves and the derived Gaussian concentration distributions, can be obtained on request from eaton@nih.gov.

## Supplementary Material

Supplementary File

## Footnotes

*Because of the relation between the delay time and supersaturation (*SI Appendix*, Fig. S3*B*), which depends on the solubility (*c_s_*), the delay time increases, and the fraction polymerized at equilibrium decreases as the solubility increases. Consequently, even though the calculated fraction polymerized rarely occurs in vivo, both the kinetics and fraction polymerized depend on the supersaturation, which results in valid correlations between clinical severity with hemoglobin composition and red cell concentration found in the various sickle syndromes (86), as first pointed out by Sunshine et al. (36). However, all of the calculations of solubility and polymer fraction for hemoglobin mixtures in ref. 86 are lower and higher, respectively, than the correct values since the equations employed did not include the effect of total hemoglobin concentration on the solubility, which had been published 6 y earlier in ref. 28; see also ref. 54.

## Change History

November 20, 2020: The PDF version of this article has been updated to correct Equation 7.
