# Supplementary material for: Allosteric control of hemoglobin S fiber formation by oxygen and its relation to the pathophysiology of sickle cell disease
Source: Proc Natl Acad Sci U S A. 2020 Jun 11;117(26):15018–27. doi: 10.1073/pnas.1922004117 (PMC7334536; doi:10.1073/pnas.1922004117)
Supplement: Supplementary File [file pnas.1922004117.sapp.pdf]

Supplementary Information for "Allosteric control of hemoglobin S fiber formation by oxygen and its relation to the patho-physiology of sickle cell disease" by Henry *et al.*

Solubility Equations

Derivation of equation (7) for solubility of hemoglobin mixtures as a function of fractional saturation of hemoglobin with oxygen in the liquid phase *at equilibrium*. Under appropriate conditions, tetrameric Hb is able to dissociate into constituent αβ dimers, which are themselves extremely stable against further dissociation into individual subunits. Once dissociated, dimers provided by each Hb homotetramer variant present in the cell may associate with dimers from other variants to produce hybrid tetramers. For our purposes, each Hb homotetramer variant which can potentially copolymerize with HbS is chemically distinguished by the identity of its β chain, and hence by the identity of its αβ dimers. We anticipate the need to distinguish overall tetramer and dimer populations by adopting a single-subscript notation to identify overall populations of specific such dimer variants; with this choice we use x_i and f_i to denote the fractional population of variant i in the solution and polymer phases, respectively. Each tetramer species consists of a pair of dimers of variant i and j; homotetramer species correspond to the case $i = j$, and hybrid heterotetramers correspond to $i \neq j$. We use x_{ij} and f_{ij} to represent the population of this tetrameric species in the solution and polymer phases, respectively.

With this notation for tetrameric species populations, the fundamental copolymerization equations of Minton (1) for mixtures of HbS with non-S hemoglobins at equilibrium, including the water activity term required by the Gibbs-Duhem relation, are

$$\frac{\gamma_s c_s}{\gamma_s^0 c_s^0}\left(\frac{a_{H_2O}}{a_{H_2O}^0}\right)^n = \frac{1}{\sum x_{ij}\varepsilon_{ij}} = \Gamma\frac{c_s}{c_s^0} \tag{S1}$$

and

$$f_{ij} = \frac{x_{ij}\varepsilon_{ij}}{\sum x_{ij}\varepsilon_{ij}} \tag{S2}$$

where c_s is the solubility, defined as the total concentration of the Hb's in the supernatant after sedimenting the fibers in an ultracentrifuge, c_s^0 is the solubility of pure unliganded HbS, the γ's are the corresponding activity coefficients, $a^o_{H_2O}$ is the activity of the water in the supernatant for pure unliganded HbS, a_{H_2O} is the activity of the water at the solubility for the mixture, and n is the number of moles of water per mole of hemoglobin in the polymer phase. The quantities ε_{ij} are the respective probabilities of incorporation of the various tetrameric species into the polymer. These quantities, along with the various fractional species populations, depend upon the fractional saturation of Hb with oxygen in the liquid phase. Expressions for the activity coefficients and for the water activity factor, both of which are functions of concentration, are given by Eaton and Hofrichter (2). In equation (S1) we have defined for notational simplicity the quantity

$$\Gamma \equiv \frac{\gamma_s}{\gamma_s^0}\left(\frac{a_{H_2O}}{a_{H_2O}^0}\right)^n \qquad \text{(S3)}$$

Under conditions in which the various initial homotetrameric species may dissociate into dimers and these dimer variants potentially reassociate into hybrid species, we must proceed by imposing mass-conservation conditions in terms of dimer populations alone. Assume that we have q distinct dimer species. If we have N total tetramers in the polymer phase, then the total number of tetramers made up of dimeric species i and j is simply $n_{ij} = f_{ij} N$. The total number of dimers of species i in the polymer is computed by simply summing over the number of dimers of that type contributed by each of the possible hybrid species, that is

$$n_i = \sum_{l \geq k} \left(\delta_{ik} + \delta_{il}\right) f_{kl} N \qquad \text{(S4)}$$

where δ_{ij} is the well known Kronecker delta symbol ($\delta_{ij} = 1$ if $i = j$ and 0 otherwise). Dividing by the total number of dimers in the polymer (= $2N$) yields the fraction of dimer species as

$$f_i = \frac{1}{2} \sum_{l \geq k} \left(\delta_{ik} + \delta_{il}\right) f_{kl} \qquad \text{(S5)}$$

The expression for overall mass conservation of Hb tetramers existing in the solution and polymer phases may be written as

$$c_s v_s + c_p v_p = c_0 \qquad \text{(S6)}$$

where c_s is as defined above, c_p is the Hb concentration in the polymer, c_0 is the total Hb concentration in the system, and v_s and v_p are the volume fractions of the solution and polymer phases, respectively. Noting that $v_s = 1 - v_p$ we have

$$v_p = \frac{c_0 - c_s}{c_p - c_s} \qquad \text{(S7)}$$

Because all total dimer populations are simply two-fold larger than the overall tetramer populations in the two phases, we can express mass conservation of the individual dimer variant i in the two phases as

$$x_i c_s \left(1 - v_p\right) + f_i c_p v_p = X_i c_0 \qquad \text{(S8)}$$

where X_i if the total fractional population of variant i, which will be the same as the initial fractional population of the homotetramer from which it is derived. Using (S7) then yields

$$x_i c_s \left(c_p - c_0\right) + f_i c_p \left(c_0 - c_s\right) - X_i c_0 \left(c_p - c_s\right) = 0 \qquad \text{(S9)}$$

Combining (S1), (S2) and (S5) then yields

$$x_i c_s\left(c_p - c_0\right) + \frac{1}{2}\Gamma\frac{c_s}{c_s^0}\sum_{l\geq k}\left(\delta_{ik} + \delta_{il}\right)\varepsilon_{kl} x_{kl}\, c_p\left(c_0 - c_s\right) - X_i c_0\left(c_p - c_s\right) = 0 \qquad \text{(S10)}$$

There is one such equation for each of the q distinct dimer species. However, because we require that $\sum_{i=1}^{q} x_i = 1$, only q-1 of the x_i are independent. Therefore, we can rewrite this as q equations in q-1 unknown x_i, plus the unknown solubility c_s.
As written, the system of equations (S10) incorporates the solution tetramer fractional concentrations x_{kl}. In order to reduce this system to manageable form, we must express these fractions in terms of the fractional concentrations of the various dimer species. In the simplest case, where we assume that all tetrameric species are equally stable, these solution tetramer fractional concentrations may be written

$$x_{ij} = \beta_{ij} x_i x_j \qquad \text{(S11)}$$

where the β_{ij} are simple binomial coefficients (β_{ij} = 2 if i = j and 1 otherwise). With this simplification, the system of equations (S10) becomes

$$x_i c_s\left(c_p - c_0\right) + \frac{1}{2}\Gamma\frac{c_s}{c_s^0}\sum_{l\geq k}\left(\delta_{ik} + \delta_{il}\right)\varepsilon_{kl}\beta_{kl} x_k x_l\, c_p\left(c_0 - c_s\right) - X_i c_0\left(c_p - c_s\right) = 0 \qquad \text{(S12)}$$

This is a system of nonlinear equations which must be solved numerically for the x_i (i=1…q-1) and c_s.

Within this model for the copolymerization, the system of equations (S10) or (S12) provides a general and exact setting for determining the solubility c_s of the mixture as a function of fractional saturation of Hb with oxygen in solution, and therefore as a function of the oxygen pressure. The crux of its practical application is the specification of the pressure-dependent copolymerization probability ε_{ij} for each tetrameric species. We make here the simplest assumption, that the variation of this probability with pressure is the same in all variants. In that case, we may write each copolymerization probability as a single pressure-dependent probability $Z(p)$ weighted by some intrinsic overall probability e_{ij} of incorporation of that variant into the polymer, that is

$$\varepsilon_{ij}\left(p\right) = e_{ij} Z\left(p\right) \qquad \text{(S13)}$$

We also assume that only Hb tetramers in the T quaternary structure are able to incorporate significantly into the polymer. Then for the MWC partition function, it is straightforward to show that

$$Z\left(p\right) = \frac{L\left(1 + K_P p\right)^4}{L\left(1 + K_T p\right)^4 + \left(1 + K_R p\right)^4} \qquad \text{(S14)}$$

where K_T and K_R are the dissociation constants for oxygen from the T state and the R state in the solution phase, respectively, and K_P is the dissociation constant for oxygen from the T state in the polymer phase; K_P is expected to be less than K_T (3).

Determination of total hemoglobin intracellular concentration distributions from red cell density distributions.

Determination of red cell density distributions from partial cumulant data. The Galacteros/Bartolucci group has gathered extensive density data using phthalate oil mixtures for the red cells of a large number of patients with sickle cell disease (4). For the present analysis, we selected 29 patients not on hydroxyurea therapy or on recent blood transfusions as evidenced by the lack of any measurable HbA. One problem in converting the density data to density distributions is that the data consists of set of 11 density values, spanning the range 1.08 g/mL to 1.15 g/mL, versus the cumulative fraction of cells with densities above each value. What makes the conversion uncertain at the low- and high-density extremes of the distribution is that less than 100% of the cells have a density greater than 1.08 g/mL and the fraction of cells with density greater than 1.15 is very significant, most frequently higher than 10%. An accurate distribution at the extremes requires that 100% of the cells have a density higher than the lowest measured density and 0% of cells have a density higher than the highest measured density.

In converting the measured cumulative distribution to a probability distribution, we therefore had to make assumptions based on previous density distribution measurements by others and to impose constraints in the fitting procedure. We assumed that there are 3 populations of cells, a low-density population corresponding to reticulocytes, a high-density population corresponding to cells that have been dehydrated from multiple sickling/usickling cycles, and the major population that is neither reticulocytes nor dense cells. The measured cumulative distribution for each patient was least-squares fitted to the cumulative distribution derived from a normalized underlying probability distribution given by the sum of three Gaussian functions with peak positions within the experimental density range. The 11 data points in each case exceeds the 9 fit parameters (three for each Gaussian component: peak position, width and amplitude) to a small enough extent that the normal fit procedure produced a range of parameter values that depended on the starting parameters, but were nevertheless consistent with the measurements. Individual sets of best-fit parameter values were highly interdependent (high covariance) in each case. We therefore adopted a Monte Carlo approach to the analysis, in which many fits were performed to the same data, with randomly selected starting parameter values. Individual fits were stabilized by imposing the constraints that (i) the peak position for the Gaussians corresponding to the least dense and most dense populations are at lower and higher densities than the peak position for the major population, respectively, (ii) the amplitude of the Gaussians for least dense and most dense populations were less than one-half the amplitude of the Gaussian corresponding to the major population, and (iii) the width of the Gaussians corresponding to the least dense and most dense populations differ by less than a factor of 1.5 from the Gaussian corresponding to the major population.

The results of the many (10,000) such fits to each patient data set, with random starting parameter values, naturally fell into various clusters distinguished by sets of parameter values and overall quality (sum of squared errors) of the fit. For each patient, the parameter values from the fits in the cluster corresponding to the smallest sum-of-squares were used to compute an average distribution,

which was taken to be the best estimate of the distribution for that patient. Moreover, repetitions of the entire process produced very similar patterns of clustering of fit parameters; as a result, repeated analyses for a given patient produced essentially identical estimated distributions. An overall average density distribution representing the full set of patients, given in Fig 5 of the main text, was computed as the simple arithmetic average of these estimated distributions for all 29 patients.

We note that applying the above procedure with distributions consisting of two Gaussians produced significantly worse fits to the individual patient data than using three.

Relation between red cell density and intracellular concentration distribution. Our next problem was to convert the density distributions to hemoglobin concentration distributions. The best data for this purpose is contained in Tables 2 and 3 in the paper by Lew et al. (5), which shows a clear linear dependence of concentration on density (Figure S1), as expected for the density of a 2 component system, as shown in Fig. S1.

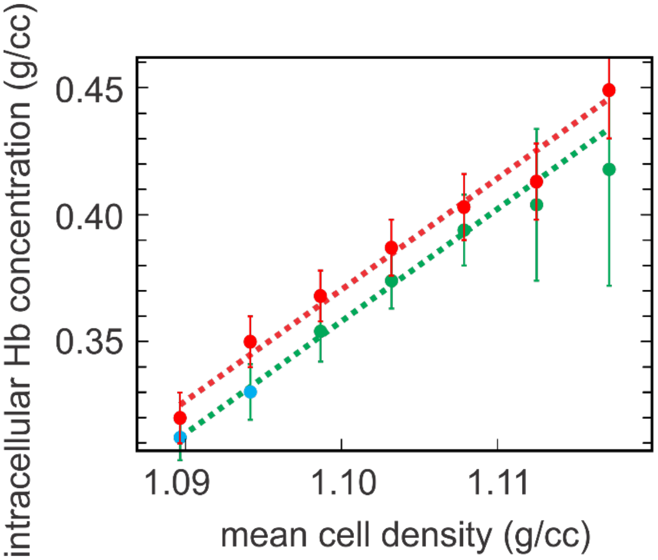

Fig. S1. Data of Lew et al. (5) for normal red cells. The green points are taken from Table 2 pf their paper, while the red points are from Table 3. The mean intracellular hemoglobin concentration (MCHC) for each density fraction was determined using the dual light scattering method of Tycko and Mohandas (6), The mean density for each fraction is the average of the reported extremes of the density range. The slope and intercept of the best least squares line, weighted by the reported uncertainties for the individual points, are 4.38 and -4.53 g/mL, respectively, for the dashed green line and 4.48 and -4.45 g/mL, respectively, for the dashed red line.

Density (ρ) is the total mass (m) divided by the total volume (V). Ignoring the membrane and considering only the two major components of the red cell, hemoglobin and its solvent consisting of water, salts, and non-hemoglobin proteins such as carbonic anhydrase, the density is given by the sum of the masses of its 2 components (m_{Hb} and m_s in grams) divided by the sum of their volumes ($m_{Hb}v_{Hb}$ and $m_s v_s$ in mL), i.e.

$$\rho = \frac{m}{V} = \frac{m_{Hb} + m_s}{m_{Hb} v_{Hb} + m_s v_s} \text{ and } c_{Hb} = \frac{m_{Hbs}}{m_{Hb} v_{Hb} + m_s v_s} \quad \text{(S15)}$$

where v_{Hb} and v_s are the partial specific volumes of the hemoglobin and its solvent, respectively and c_{Hb} is the intracellular concentration of hemoglobin. After rearrangement, the hemoglobin concentration as a function of cell density is given by

$$c_{Hb} = \frac{\rho v_s - 1}{v_s - v_{Hb}} \quad \text{(S16)}$$

To measure the partial specific volume of the solvent for hemoglobin by emptying only the hemoglobin from a red cell without any changes in red cell volume or content is not yet possible. However, it is possible to reduce the number of fitting parameters in the linear-squares fit to the data of Lew *et al* in Figure S1 from two to one by determining v_{Hb}, which is described below.

Using the measured value of 0.76 mL/g, the one component fit using eqn. S(16) in which only v_s was varied, yields a density for the average of the 2 determinations of the hemoglobin solvent ($=1/v_s$) of 1.017 g/mL, possibly a physically unreasonable value given that estimated density from carbonic anhydrase, the most abundant non-hemoglobin protein in the red cell, at a concentration of 0.0017 g/ mL (60 μM??, (7) assumed to have the same partial specific volume of 0.76 g/ mL as hemoglobin, and the salts estimated as sodium chloride in normal saline, with a partial specific volume that is the reciprocal of its 1.0046 g/mL density, is only 1.033. With the values for the partial specific volumes of 0.76 mL/g for v_{Hb} and 0.983 mL/g (= 1/1.017) for v_s, we can calculate the hemoglobin concentration from the density using the simple linear equation:

$$c_{Hb}(g/cc) = \frac{v_s}{v_s - v_{Hb}}\rho - \frac{1}{v_s - v_{Hb}} = 4.41\rho - 4.49$$

At a density of 1.0905 g/mL, density/[Hb] calculated from equation S16 is 3.43, while the values from the previous studies mentioned in the text are 3.61 and 3.02 at densities of 1.091 and 1.090. respectively (8, 9).

Determination of the partial specific volume of hemoglobin at the high concentrations found in red cells. Partial specific volumes of hemoglobin have been determined, but only concentrations of approximately 10 mg/ml, more than 30-fold less than the concentrations found in red cells (10). Given the sensitivity of the dependence of concentration on red cell and solvent partial specific volumes, we decided to determine the partial specific volume at hemoglobin concentrations found in red cells. Purified HbA was prepared from blood of W.A.E. Packed cells from 10 ml of blood were washed three times with ice cold phosphate buffered saline (PBS, 1.7 mM KH_2 PO_4, 5 mM Na_2HPO_4, 150 mM NaCl, pH 7.4). Between gently suspending cells in PBS, they were harvested by centrifugation at 2000 rpm (Beckmann Allegra 6KR swinging bucket rotor GH-3.8) for 7 min. Packed cells (4 ml) kept on ice were lysed by suspension in 16 ml ice-cold deionized water followed by centrifugation at 30,600 x g for 30 min at 4 °C. The supernatant was centrifuged a second time, and aliquots of the supernatant were stored at -80 °C until further use.

HbA was purified on a XK-26 column (GE Healthcare) packed with 75 ml Q Sepharose high performance resin (GE Healthcare, Catalog number 17-1014-01). The lysate (4 ml) was diluted 4-fold in 20 mM Tris-HCl at pH 8.5 (buffer A), passed through the column, and washed in buffer A. Bound HbA was eluted in a linear gradient of 0-150 mM NaCl in 4 column volumes at a flow rate of 8 ml/min. Peak fractions of HbA were pooled, concentrated using 30k cut-off Amicon centrifugal filters and dialyzed extensively against 0.1 M sodium phosphate buffer, pH 7 HbA concentration was determined using the Drabkin's reagent (Sigma-Aldrich, Catalog number D5941-6V) at 540 nm of the CN-metHb form and the extinction coefficient per heme of 10,990 $M^{-1}cm^{-1}$ used by DeMoll et al.(10).

Density measurements were made with an Anton Paar DMA5000 precision density meter at 20 °C. Before each set of measurements, a density check and adjustment were carried out using dry air (corrected for atmospheric pressure) and ultra-pure, degassed, Milli-Q water. Required dilutions prepared from HbA stock solution were equilibrated to room temperature. For each set of measurements, the density of the most concentrated solution was first determined in duplicate, and the sample cell was subsequently washed with 0.1 M sodium phosphate until the flow-through was no longer colored, followed by water and acetone. The cell was dried, and an air density check was carried out. This procedure was repeated on dilutions of the stock solution similarly, followed by triplicate density measurements of the dialysis buffer for that sample. Aliquots of each sample were kept, and their concentration measured again using the colorimetric method of Drabkin's. Separate measurements were carried out on samples in the 2.78 – 44.5 mg/mL (measurement M1), 9.67 – 79.4 mg/mL (measurement M2), and 207.3 – 357.1 mg/mL (measurement M3) concentration ranges. Only single measurements were obtained for the highest concentration set.

Solution density measurements plotted as a function of the hemoglobin concentration provide the density increment, $(\partial\rho/\partial c)$, which is related to the apparent partial specific volume, $\bar{v}$, as follows: $(\partial\rho/\partial c) = (1 - \bar{v}\rho_o)$, where ρ_o is the buffer density. Plots of solution density versus hemoglobin concentration for measurements M1 and M2 were linear, returning best-fit slopes equivalent to an effective partial specific volume of 0.760 ± 0.002 mL/g, and best-fit intercepts equal to the measured buffer density (within the error of the method). A similar plot for measurement M3 returns an effective partial specific volume of 0.764 ± 0.003 mL/g, demonstrating that the effective partial specific volume does not depend on the protein concentration. In all cases, the best-fit line returned a coefficient of determination, r^2, of 0.9994 or larger.

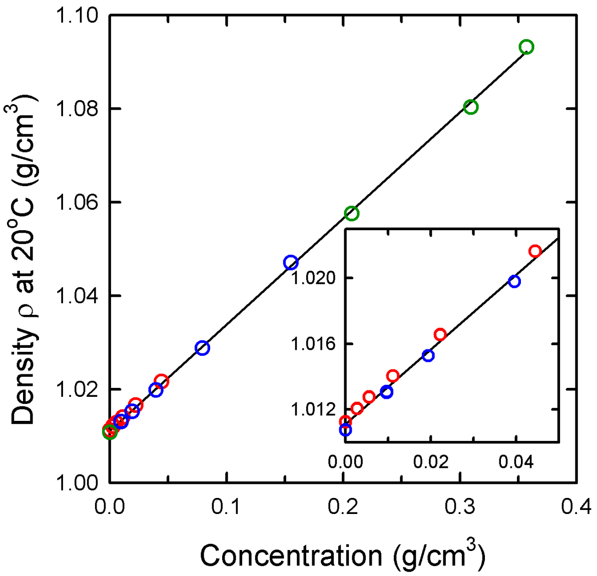

Fig. S2. Density as a function of concentration for hemoglobin solutions in 0.1 M sodium phosphate pH 7.0. Colored symbols represent separate experiments at different concentration ranges corresponding to: red 2.78 – 44.5 mg/mL; blue 9.67 – 79.4 mg/mL; and green 207.3 – 357.1 mg/mL. The best-fit line for all data sets is shown. Note that the lowest concentration range had a measured buffer density of 1.01125 g/cm^3, whereas the two highest concentration ranges had a measured buffer density of 1.01076 and 1.01074 g/cm^3. These small density differences are reflected in the inset showing the density as a function of concentration in the 2.78 – 44.5 mg/mL range.

Derivation of Szabo equation for calculating sickling times.

When the homogeneous nucleation rate is small compared to the heterogeneous nucleation rate, as is the case for the delay times comparable to the seconds time scale for transit of red cells through the microcirculation, the amount of of polymerized hemoglobin (Δ) increases exponentially (2, 11), i.e.

$$\Delta(t) = \frac{1}{2} A \exp(Bt) \qquad \text{(S14)}$$

Equating the delay time (t_d) to the tenth time ($t_{1/10}$), i.e. the time at which one-tenth the concentration of polymerized hemoglobin that is formed at equilibrium (Δ(▯)), the delay time is given by (2, 11)

$$t_d = t_{1/10} = \frac{1}{B} \ln\left(\frac{\Delta(\infty)}{5A}\right) = \frac{C}{B}, \qquad \text{(S15)}$$

where the constant C is $\ln\left(\Delta(\infty)/5A\right)$. As the saturation of HbS with oxygen decreases with time, the delay time also decreases. Treating the parameter B as a time-dependent rate coefficient and the parameter A as time-independent because of the much lower sensitivity of the delay time to A, the concentration of polymerized hemoglobin is now given by

$$\Delta(t) = \frac{1}{2} A \exp\left(\int_0^t B(\tau) d\tau \right) \qquad \text{(S16)}$$

The concentration of polymerized hemoglobin is $\sim \Delta(\infty)/10$, when the upper limit of the integral is the delay time for the cell, i.e. the sickling time

$$\int_0^{t_{sickle}} B(\tau) d\tau = C \qquad \text{(S18)}$$

Substituting $\frac{C}{t_d(\tau)}$ for $B(\tau)$ from eqn. (S15),

$$\int_0^{t_{sickle}} \frac{d\tau}{t_d(\tau)} = 1 \qquad \text{(S19)}$$

which shows that, knowing the time dependence of the delay time, the sickling time (t_{sickle}) is given by the time at which the integral = 1.

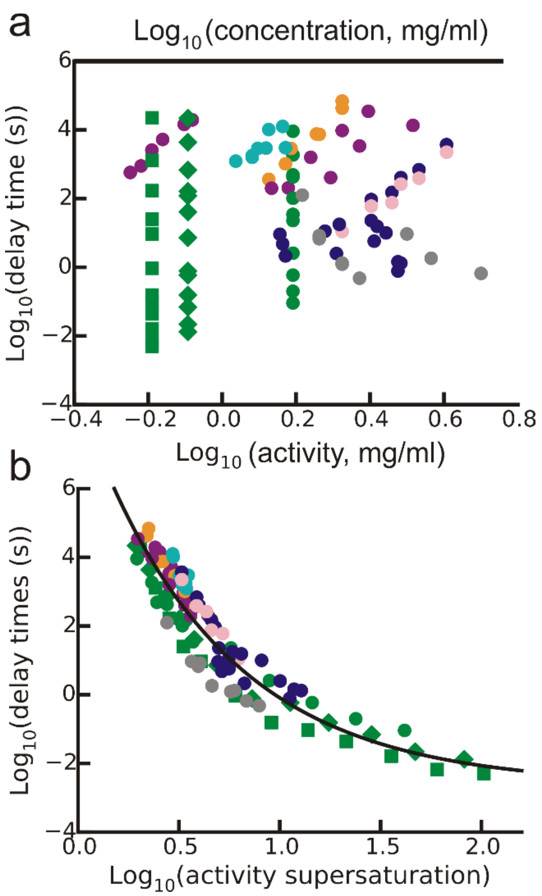

Fig. S3. Universal relation between delay time and supersaturation. (a) Delay time versus solubility (the concentration of HbS in the liquid supernatant after fibers are sedimented) and the activity (supernatant concentration multiplied by activity coefficient from equation (2). Unlike most chemical reactions for which the rate increases as the stability of the products relative to the reactants increases (linear free energy relations), there is no correlation at all between the delay time and the stability of the fiber as determined from the solubility. (b) However, when the delay time is plotted versus the ratio of the initial activity prior to fiber formation to the activity in the liquid phase (the supernatant), the data collapse onto a single universal curve, no matter how the solubility is varied (changes in temperature, carbon monoxide concentration, dilution of HbS with non-S hemoglobins, etc) (12). The continuous curve ($y = a\ \exp(-bx) + c$) is a least-squares fit to the data, where y is $\log_{10}$(delay time), x is the activity supersaturation, $a = 11.2 \pm 0.5$, $b = 1.49 \pm 0.20$, $c = -2.64 \pm 0.48$.

Table S1. Hemoglobin composition of 16 NIH patients on hydroxyurea

	HbF (%)	HbA2 (%)	HbS (%)
1	26.9	4.0	69.1
2	26.5	3.7	69.8
3	22.8	3.9	73.3
4	21.7	3.8	74.5
5	14.7	4.3	81.0
6	12.6	4.5	82.9
7	13.8	4.6	81.6
8	8.3	4.4	87.3
9	19.3	3.9	76.8
10	13.2	4.3	82.5
11	9.4	4.0	86.6
12	10.0	4.5	85.5
13	9.5	4.5	86.0
14	12.6	5.8	81.6
15	21.9	3.9	74.2
16	16.8	4.9	78.3

Table S2. Hemoglobin composition of 29 Henri-Mondor patients not on hydroxyurea

	HbF (%)	HbA2 (%)	HbS (%)
1	9.0	3.7	87.3
2	13.5	3.5	83.0
3	3.0	3.6	93.4
4	3.0	3.6	93.4
5	15.5	2.8	81.7
6	4.3	4.8	90.9
7	11.0	4.3	84.7
8	9.4	4.2	86.4
9	3.0	4.1	92.9
10	4.7	4.2	91.1
11	7.8	3.4	88.8
12	0.8	3.7	95.5
13	12.0	3.8	84.2
14	2.5	4.4	93.1
15	7.0	4.0	89.0
16	11.0	4.5	84.5
17	4.0	4.0	92.0
18	6.0	3.7	90.3
19	8.5	4.4	87.1
20	2.0	4.2	93.8
21	21.0	2.6	76.4
22	7.0	4.0	89.0
23	3.5	4.2	92.3
24	6.0	5.5	88.5
25	17.0	3.5	79.5
26	3.4	4.4	92.2
27	7.0	4.0	89.0
28	15.0	3.0	82.0
29	11.0	3.8	85.2

Details of calculations for figures in main text.

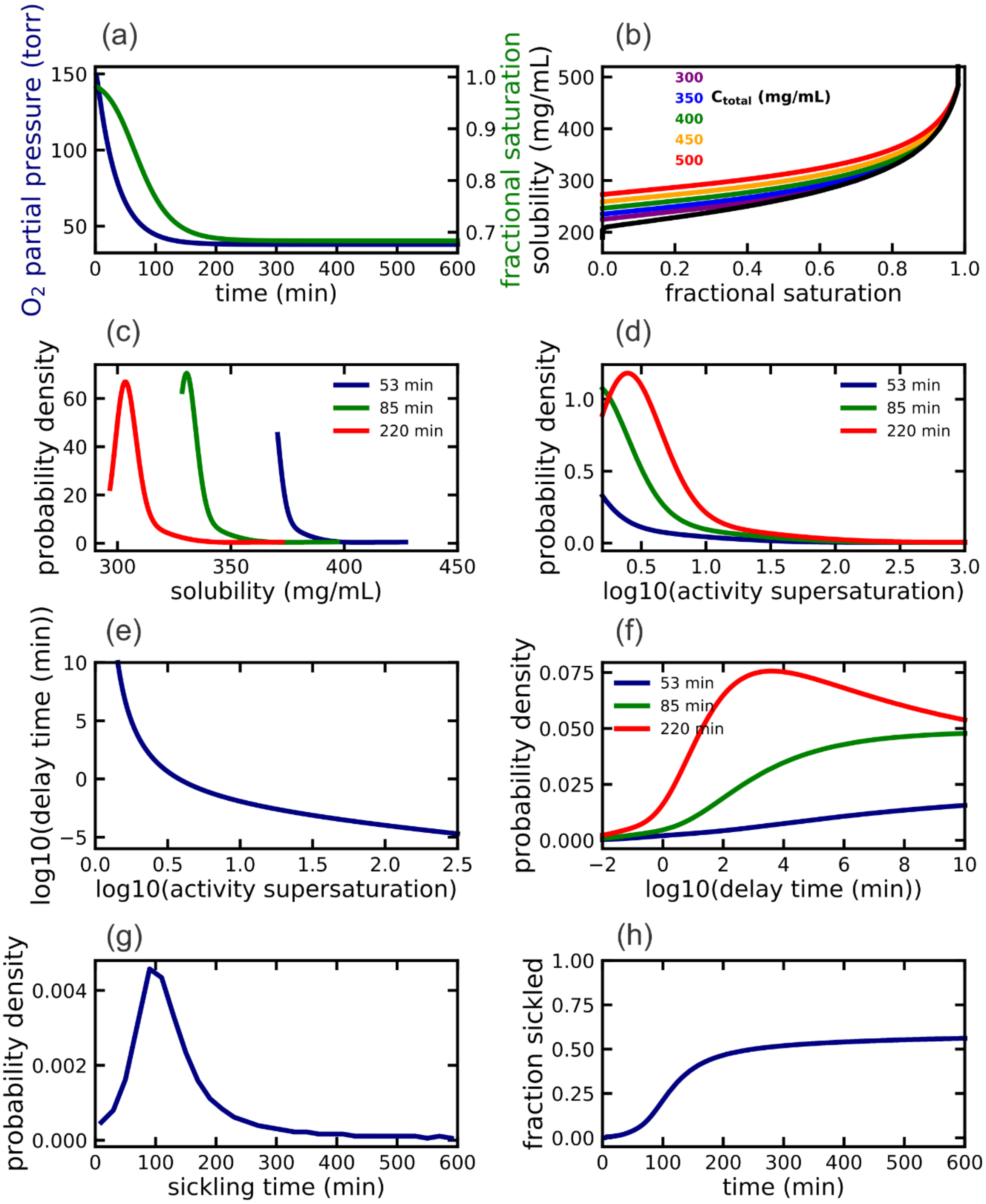

Figure S4. Calculation of fraction sickled versus time in Figure 9 for slow *in vitro* deoxygenation to 95% nitrogen and 5% oxygen for red cells from SS patients on hydroxyurea. (a) (blue curve) Oxygen partial pressure in contact with SS cells at the bottom of wells of 384 well plate as a function of time as oxygen diffuses away, calculated as in Dunkelberger et al. (13), and (green curve) the corresponding fractional saturation with oxygen of the sickle hemoglobin in the liquid phase calculated from equation (3) with MWC

parameters K_T = 0.0093 torr^{-1}, K_R = 1.04 torr^{-1}, L = 5.6x10^5. Oxygen binding and dissociation occurs on the sub-second time scale, so the fractional saturation is determined solely by the oxygen pressure. (b) Solubility vs saturation for a hemoglobin mixture of 88% HbS and 12% HbF + HbA2, the average composition of the red cells in the red cells of the 16 SS patients. The copolymerization probability for the Hb f and HbA2 homotetramers is zero, while the copolymerization probabilities for the S/F and S/A2 hybrid tetramers is 0.1. The solubilities are calculated for the range of total Hb intracellular concentrations from 250 mg/mL to 500 mg/mL every 50 mg/mL (Fig. 5). (c) The distribution of solubilities at 3 different time points during deoxygenation. (d) The distribution of activity supersaturation ratios at the same time points. (e) The universal curve relating delay time and activity supersaturation (12). (f) Distribution of delay times calculated from panels (d) and (e). (g) Distribution of sickling times at the 3 times points calculated from equation (8). (h) Average fraction sickled vs time for cells of 16 SS patients being treated with hydroxyurea.

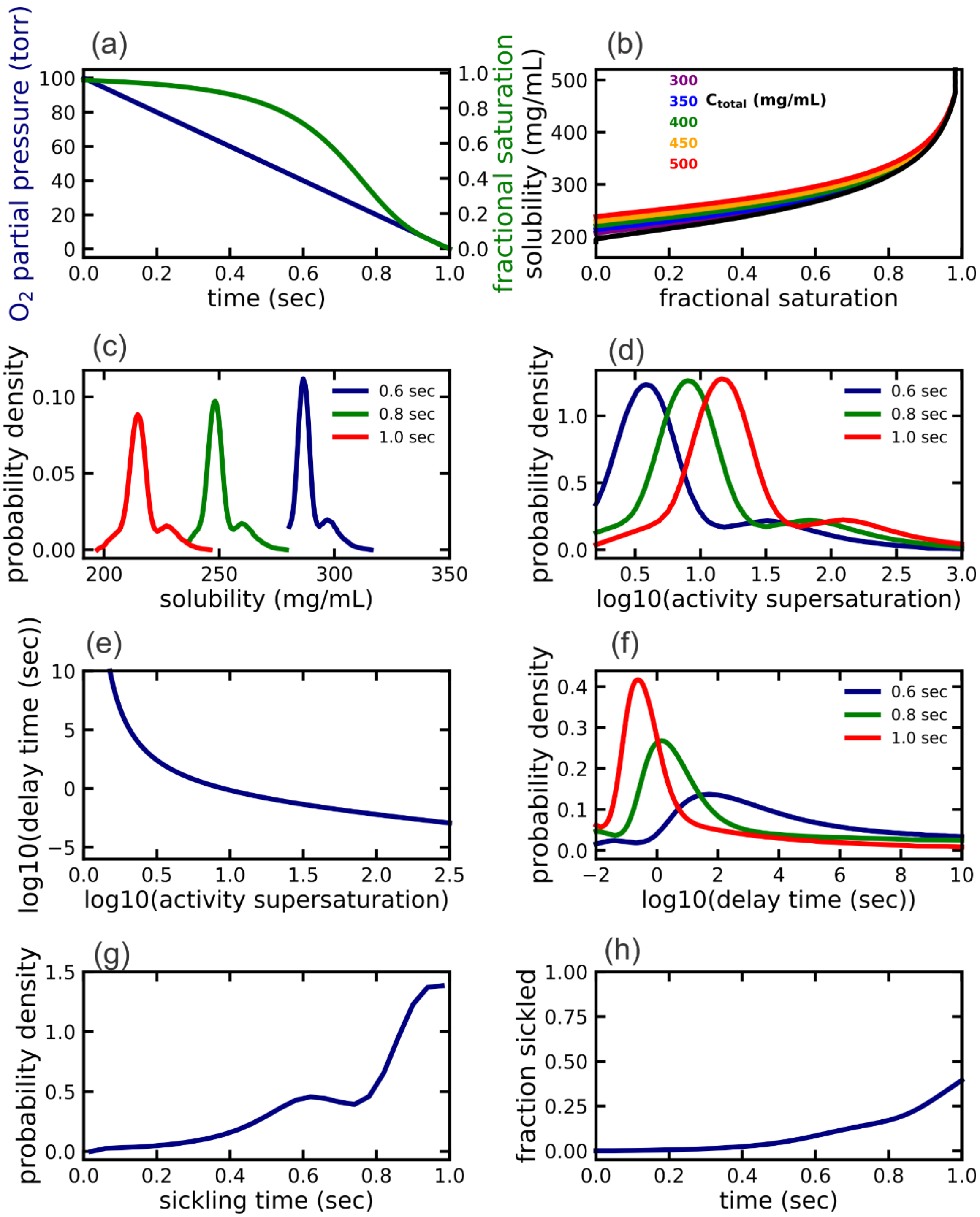

Figure S5. Calculation of fraction sickled vs time for rapid *in vivo* deoxygenation of SS cells for Figure 11 of main text. (a) (blue curve) Assumed oxygen partial pressure as a function of time and (green curve) the corresponding fractional saturation with oxygen of the sickle hemoglobin in the liquid phase calculated from equation (3) with MWC parameters K_T = 0.0093 torr^{-1}, K_R = 1.04 torr^{-1}, L = 5.6x10^5. We assume that oxygen binding and dissociation is rapid enough that the fractional saturation is determined solely by the oxygen pressure. We also assumed that tetramer-dimer dissociation and reassociation is sufficiently slow, so the composition of the 3 tetramers in the liquid phase is fixed by the composition when fully oxygenated. The equations used in these calculations are those given by Dunkelberger, *et al*.(13). There is only a small

difference in the solubility as a function of oxygen saturation compared to what is obtained from equations 7 and 8 (equation S12). (b) Solubility vs saturation for a hemoglobin mixture of 88% HbS and 12% (HbF + HbA2), the average composition of the red cells from SS patients not on hydroxyurea. The solubilities are calculated for the range of total Hb intracellular concentrations from 250 mg/mL to 500 mg/mL every 50 g/mL (Fig. 5). (c) The distribution of solubilities at 3 different time points in the deoxygenation. (d) The distribution of activity supersaturation ratios at the same time points. (e) The universal curve relation delay time and activity supersaturation (12) (see Fig. S2). (f) Distribution of delay times calculated from panels (d) and (e). (f) Distribution of sickling times at the 3 times points calculated from equation (8). (g) Fraction sickled vs time.

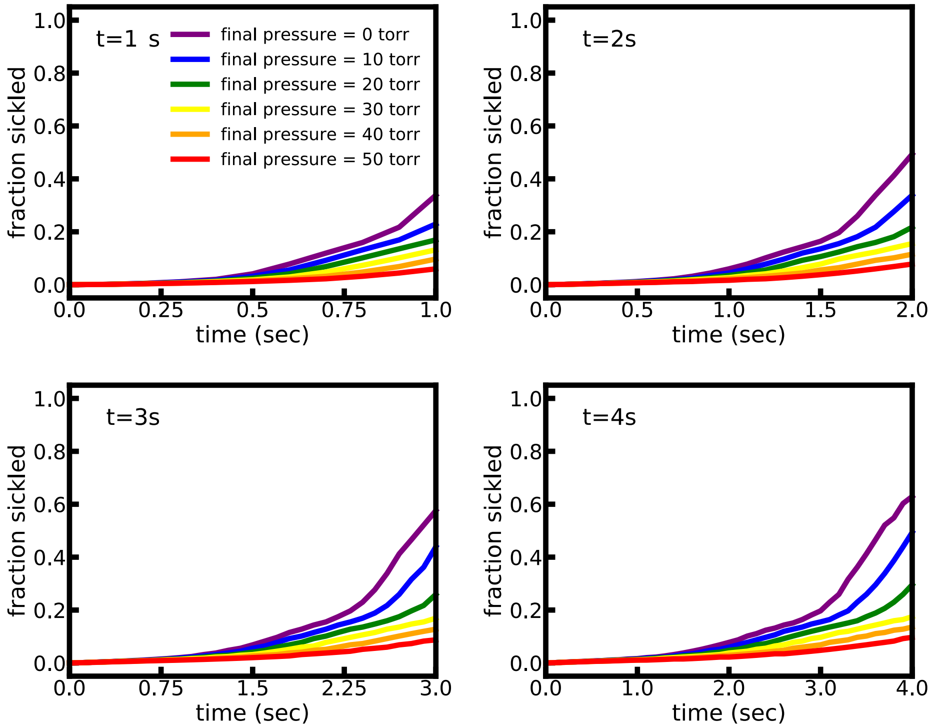

Figure S6. Calculated *in vivo* SS kinetics for Figure 10 of main text. Fraction sickled vs time for average SS composition and concentration distribution from red cells of 29 patients not treated with hydroxyurea (see Table S2 for Hb compositions) for a range of times between 1 and 4 seconds for oxygen pressure to linearly decrease from 100 torr to 6 different final pressures.

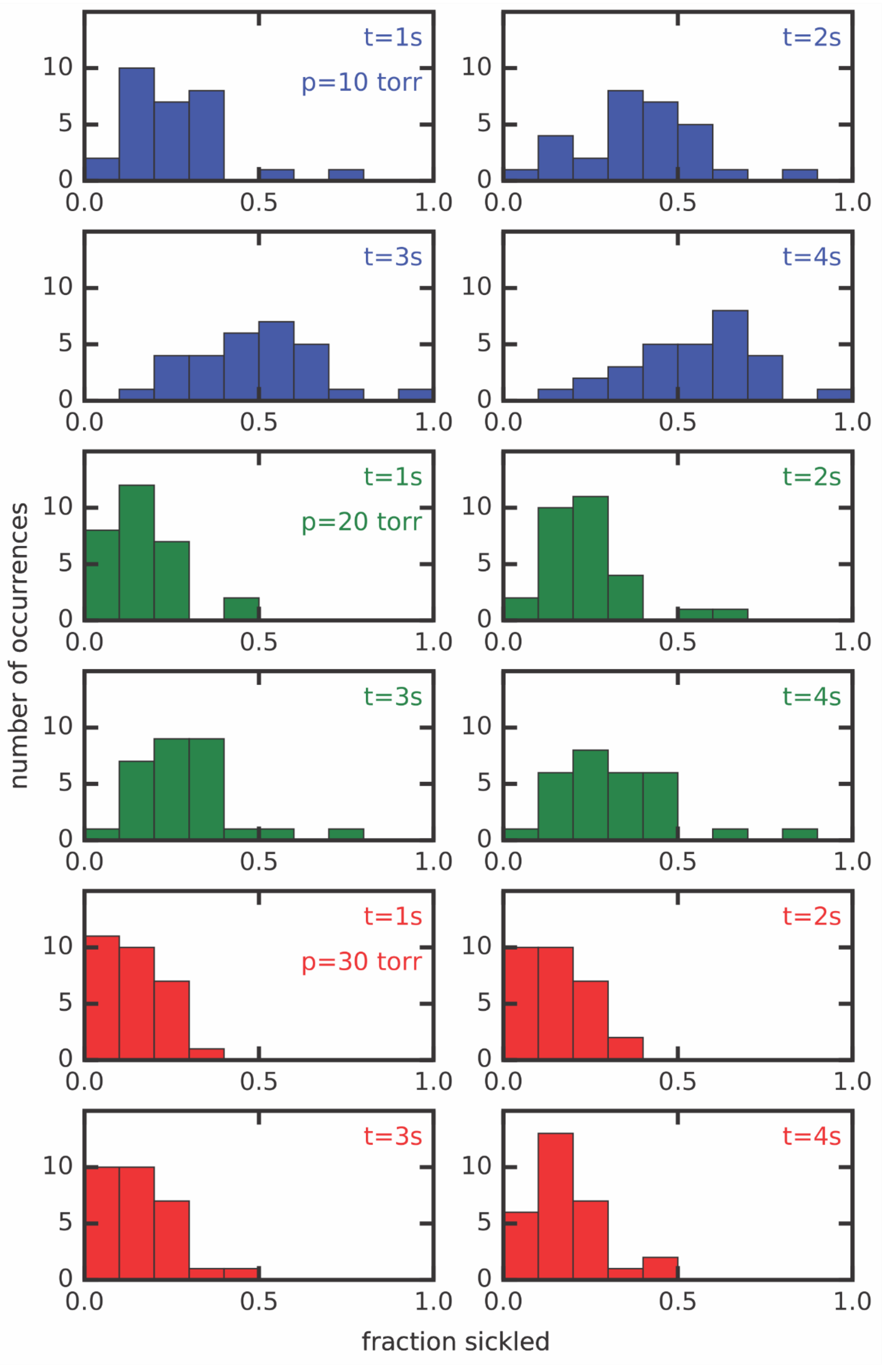
t=1s
p=10 torr
t=2s
t=3s
t=4s
t=1s
p=20 torr
t=2s
t=3s
t=4s
t=1s
p=30 torr
t=2s
t=3s
t=4s
number of occurrences
fraction sickled

Figure S7. Calculated distribution of the fraction sickled at final pressures of 10, 20, and 30 torr for 29 SS patients not on hydroxyurea.

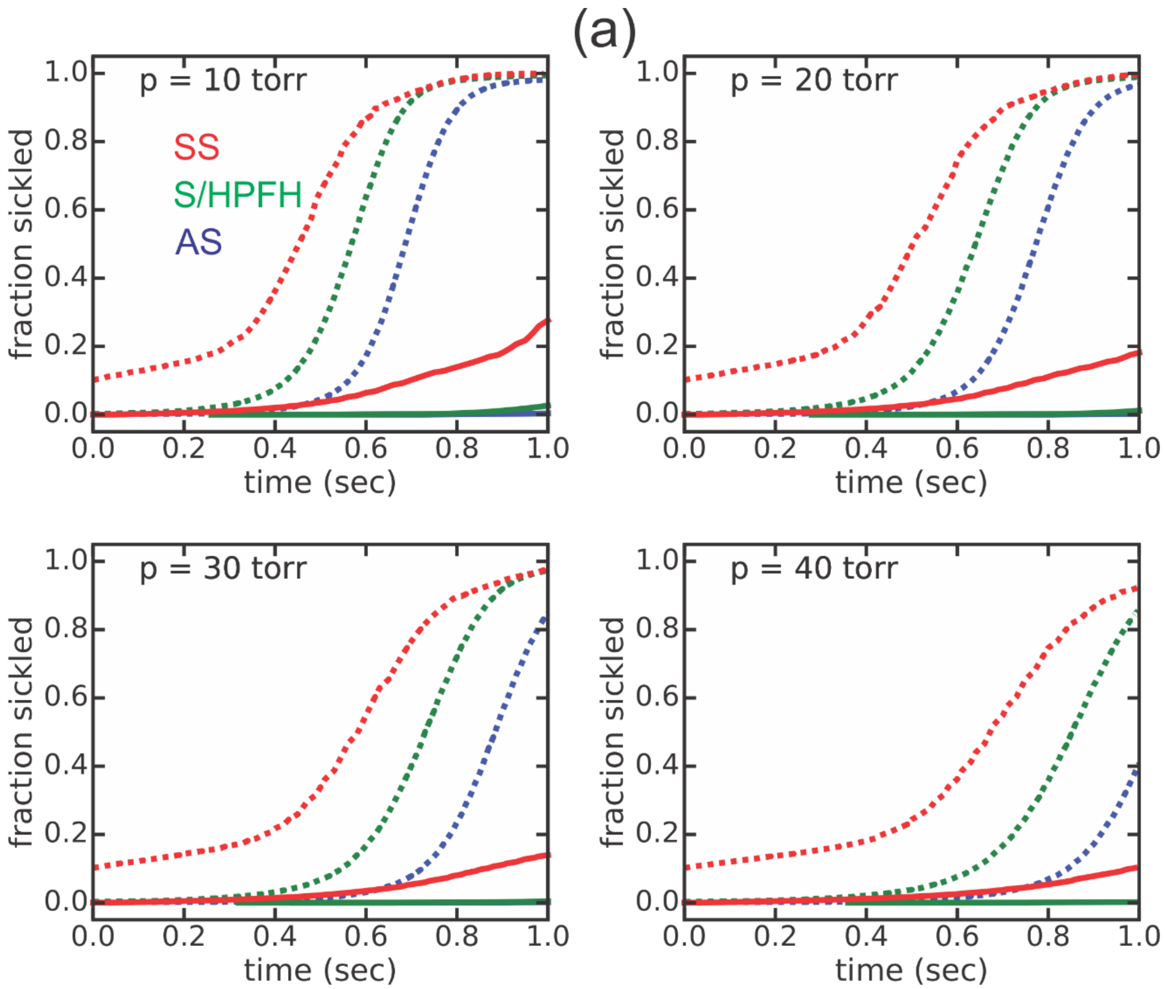

Figure S8. Comparison of calculated *in vivo* SS (red), S/PHFH (green), and AS (blue) sickling kinetics together with fraction sickled at equilibrium. Fraction sickled vs time induced by a one-second linear decrease in oxygen pressure for average hemoglobin composition and average concentration distribution for cells from SS patients not being treated with hydroxyurea (Figures 11-13), sickle trait donors with average composition of 38% HbS, 58% HbA and 4% HbA2 and for red red cells having a hemoglobin composition of 30% HbF and 70% HbS as found in the compound heterozygous condition of S/HPFH.

References

1. Minton AP (1977) Non-ideality and thermodynamics of sickle cell hemoglobin gelation. ***J. Mol. Biol.*** 110(1):89-103.
2. Eaton WA & Hofrichter J (1990) Sickle cell hemoglobin polymerization. *Adv. Prot. Chem.* 40:63-279.
3. Henry ER*, et al.* (2015) Experiments on hemoglobin in single crystals and silica gels distinguish among allosteric models. *Biophys. J.* 109(6):1264-1272.

4. Bartolucci P*, et al.* (2012) Erythrocyte density in sickle cell syndromes is associated with specific clinical manifestations and hemolysis. *Blood* 120(15):3136-3141.
5. Lew VL, Raftos JE, Sorette M, Bookchin RM, & Mohandas N (1995) Generation of normal human red cell volumes, hemoglobin content. and membrane area distributions by birth or regulation. *Blood* 86(1):334-341.
6. Mohandas N*, et al.* (1986) Accurate and independent measurement of volume and hemoglobin concentration of individual red cells by laser light scattering. *Blood* 68(2):506-513.
7. Dodgson SJ, Forster RE, Sly WS, & Tashian RE (1988) Carbonic anhydrase activity of intact carbonic anhydrase II deficient human erythrocytes. *J. App. Physiol.* 65(4):1472-1480.
8. Hosseini P*, et al.* (2016) Cellular normoxic biophysical markers of hydroxyurea treatment in sickle cell disease. *Proc, Natl. Acad. Sci. USA* 113(34):9527-9532.
9. Schwartz RS, Musto S, Fabry ME, & Nagel RL (1998) Two distinct pathways mediate the formation of intermediate density cells and hyperdense cells from normal density sickle red blood cells. *Blood* 92(12):4844-4855.
10. DeMoll E, Cox DJ, Daniel E, & Riggs AF (2007) Apparent specific volume of human hemoglobin: Effect of ligand state and contribution of heme. *Anal. Bioche.* 363(2):196-203.
11. Ferrone FA, Hofrichter J, & Eaton WA (1985) Kinetics of sickle hemoglobin polymerization 2. A double nucleation mechanism. *J. Mol. Biol.* 183(4):611-631.
12. Cellmer T, Ferrone FA, & Eaton WA (2016) Universality of supersaturation ratio in protein fiber formation. *Nature Struct. Mol. Biol.* 23:459-471.
13. Dunkelberger EB, Metaferia B, Cellmer T, & Henry ER (2018) Theoretical simulation of red cell sickling upon deoxygenation based on the physical chemistry of sickle hemoglobin fiber formation. *J. Phys. Chem. B* 122(49):11579-11590.
